# SDS-PAGE analysis of Aβ oligomers is disserving research into Alzheimer´s disease: appealing for ESI-IM-MS

**DOI:** 10.1038/srep14809

**Published:** 2015-10-09

**Authors:** Rosa Pujol-Pina, Sílvia Vilaprinyó-Pascual, Roberta Mazzucato, Annalisa Arcella, Marta Vilaseca, Modesto Orozco, Natàlia Carulla

**Affiliations:** 1 Institute for Research in Biomedicine (IRB Barcelona), Baldiri Reixac 10, Barcelona 08028, Spain; 2 Joint IRB-BSC Research Program in Computational Biology, Institute for Research in Biomedicine (IRB Barcelona), Baldiri Reixac 10, Barcelona 08028, Spain; 3 Mass Spectrometry Core Facility, Institute for Research in Biomedicine (IRB Barcelona), Baldiri Reixac 10, Barcelona 08028, Spain; 4 Department of Biochemistry and Molecular Biology, University of Barcelona, Diagonal 647, Barcelona 08028, Spain

## Abstract

The characterization of amyloid-beta peptide (Aβ) oligomer forms and structures is crucial to the advancement in the field of Alzheimer´s disease (AD). Here we report a critical evaluation of two methods used for this purpose, namely sodium dodecyl sulfate polyacrylamide gel electrophoresis (SDS-PAGE), extensively used in the field, and ion mobility coupled to electrospray ionization mass spectrometry (ESI-IM-MS), an emerging technique with great potential for oligomer characterization. To evaluate their performance, we first obtained pure cross-linked Aβ40 and Aβ42 oligomers of well-defined order. Analysis of these samples by SDS-PAGE revealed that SDS affects the oligomerization state of Aβ42 oligomers, thus providing flawed information on their order and distribution. In contrast, ESI-IM-MS provided accurate information, while also reported on the chemical nature and on the structure of the oligomers. Our findings have important implications as they challenge scientific paradigms in the AD field built upon SDS-PAGE characterization of Aβ oligomer samples.

Aβ oligomers, species that form early during Aβ aggregation, are considered the pathogenic molecular form of Aβ in AD^1^. Consequently, they have been singled out as a target to treat this disease^2^. However, the characterization of Aβ oligomers is challenging because they are heterogeneous—comprising a range of aggregation states—and because they form transiently—evolving as a function of time^3^,^4^. In spite of these difficulties, different approaches and techniques have been developed to characterize them^5^,^6^,^7^,^8^,^9^,^10^,^11^,^12^. One such approach relies on the production of cross-linked oligomers by means of the photo-induced cross-linking of unmodified proteins (PICUP) reaction, and their subsequent analysis by SDS-PAGE (Fig. 1)^5^,^13^. Aβ1-40 (Aβ40) and Aβ1-42 (Aβ42) are the two principal forms of Aβ, differing by only two hydrophobic residues at the C-terminus. Analysis of PICUP cross-linked Aβ40 and Aβ42 samples by SDS-PAGE revealed that Aβ40 oligomerizes through dimers up to tetramers while Aβ42 does so mainly through pentamers and hexamers^5^,^13^. Since Aβ42 has been shown to have a more prominent role in AD^14^,^15^, PICUP/SDS-PAGE analysis led to the conclusion that pentamers and hexamers constituted the basic building blocks for Aβ aggregation^5^,^13^.

The importance of pentamers and hexamers in Aβ aggregation has become a scientific paradigm in the field. For example, according to the Web of Science, there are six key papers^7^,^8^,^9^,^16^,^17^,^18^, frequently cited together (more than one thousand times), that constitute the foundational core for the research front entitled “Aβ oligomers, fibrils and AD”. Three of these six papers are based directly^16^,^18^ or indirectly^7^ on the conclusions derived from PICUP/SDS-PAGE analysis. However, SDS-PAGE analysis might be biased since SDS has been suggested to affect the oligomerization state of Aβ, especially that of Aβ42^8^,^19^,^20^. Given the potential artifacts of SDS, it is critical to evaluate the usefulness of SDS-PAGE to characterize Aβ oligomers to have a criteria with which to revise the conclusions derived from its use. For the purpose of this evaluation, it is important to consider new techniques that are not dependent on SDS. ESI-IM-MS is an emerging method with great potential for the characterization of oligomers^7^,^10^,^21^,^22^,^23^,^24^. ESI-IM-MS offers the possibility to resolve heterogeneous oligomer samples on the basis of differences in the order and/or in the structure of the oligomers present in a sample. Moreover, the time scale of IM experiments (tens of ms) is much shorter than that of conventional structural techniques (s to hrs), thus making them ideal for the structural characterization of heterogeneous and dynamic samples such as Aβ oligomers. Furthermore, since ESI-MS allows the preservation of many non-covalent interactions within complexes^25^, even several involving a reduced interaction surface^26^,^27^,^28^,^29^,^30^, ESI-IM-MS emerges as a suitable method for the characterization of not only cross-linked oligomers but also non-covalent ones.

Here we provide a critical evaluation of SDS-PAGE and ESI-IM-MS for the characterization of Aβ oligomers. To this end, we first developed a protocol to obtain pure PICUP cross-linked (CL) Aβ40 and Aβ42 oligomers of defined order. Using these samples, we unequivocally established that SDS-PAGE leads to artifacts in determining the order and distribution of Aβ42 oligomers On the other hand, ESI-IM-MS was further established as a reliable technique through which to characterize the order, distribution, chemical modifications, and structure of both covalently and non-covalently linked Aβ oligomers. Our results have important implications as they challenge previous scientific paradigms in the field built upon results obtained through the SDS-PAGE characterization of Aβ oligomers. In this context, we demonstrate that pentamers and hexamers are artifacts of SDS-PAGE analysis. Moreover, we identify Aβ40 and Aβ42 dimers and trimers, adopting a globular structure and lacking defined secondary structure, as the earliest forms to be considered in the design of therapeutic strategies targeting Aβ oligomerization.

## Results

### PICUP and SDS-PAGE apparently indicate that Aβ40 and Aβ42 oligomerize through distinct pathways

**Figure 1:**
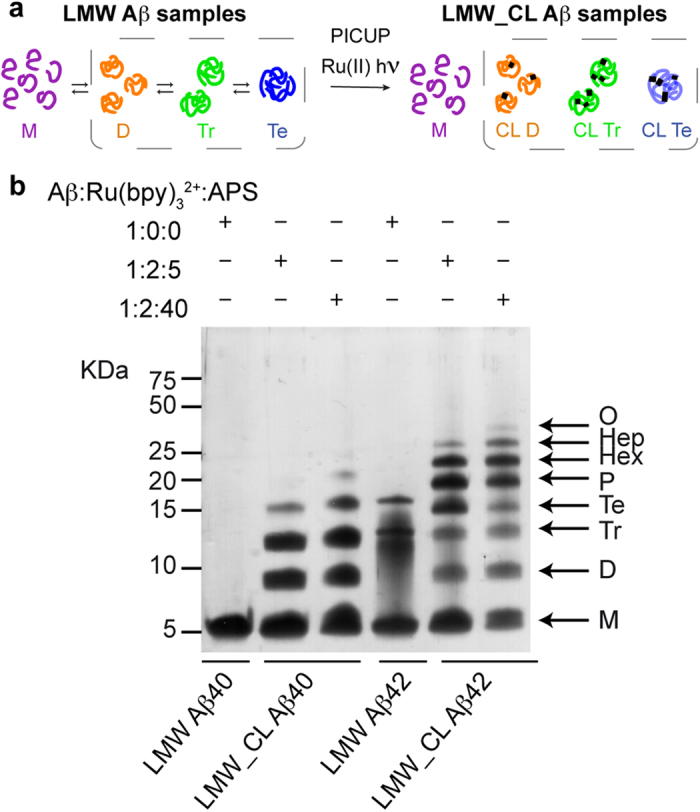
PICUP and SDS-PAGE analysis apparently indicate that Aβ40 and Aβ42 oligomerize through distinct pathways. (**a**) LMW Aβ samples contain monomers in equilibrium with low order Aβ oligomers (left). Synthetic LMW_CL Aβ samples are obtained by subjecting their LMW counterparts to PICUP (right). (**b**) Characterization of LMW and LMW_CL Aβ40 and Aβ42 oligomer distribution by SDS-PAGE analysis. LMW_CL Aβ40 and Aβ42 samples were prepared using two different Aβ/Ru(bpy)_3_^2+^/APS ratios, namely 1:2:5 and 1:2:40.

Following previously described protocols^31^,^32^, we obtained Aβ40 and Aβ42 samples in their lowest aggregation state using size exclusion chromatography (SEC). We refer to these samples as low molecular weight (LMW) Aβ40 and Aβ42 (Fig. 1a), as various techniques have shown that they comprise monomers in rapid equilibrium with low order oligomers^31^. To freeze this dynamic equilibrium, LMW Aβ samples were cross-linked by means of PICUP^5^,^13^. We refer to these samples as LMW cross-linked (LMW_CL) Aβ40 and Aβ42 (Fig. 1a). Initially, we used previously described conditions—an Aβ/tris(bipyridyl) Ru(II) complex (Ru(bpy)_3_^2+^)/ammonium persulfate (APS) ratio of 1:2:40—to obtain LMW_CL Aβ40 and Aβ42^5^,^13^,^33^. Analysis of these samples by SDS-PAGE and silver staining reproduced previous results described in the literature (Fig. 1b). Uncross-linked LMW Aβ40 ran with a molecular weight consistent with that of monomers. LMW_CL Aβ40 presented an oligomer distribution characterized by monomers through to pentamers—displaying decreasing intensity with increasing oligomer order. Uncross-linked Aβ42 produced predominantly three bands consistent with monomers, trimers and tetramers. LMW_CL Aβ42 ran as monomers through to trimers—displaying decreasing intensity with increasing oligomer order—and as a Gaussian-like distribution comprising tetramers through to octamers, with a maximum at pentamers and hexamers. These results apparently support the hypothesis from Teplow and co-workers that Aβ40 and Aβ42 oligomerize through distinct pathways^5^,^13^.

To determine the homogeneity of the LMW_CL Aβ samples, we analyzed LMW_CL Aβ40 by reversed phase high-pressure liquid chromatography (RP-HPLC) and LC coupled to high resolution MS (LC-HRMS). This analysis revealed that an Aβ/Ru(bpy)_3_^2+^/APS ratio of 1:2:40 led to various degrees of oxidized byproducts (Supplementary Fig. S1a and Table S1). We then optimized PICUP conditions and found that an Aβ/Ru(bpy)_3_^2+^/APS ratio of 1:2:5 largely overcame the formation of these byproducts (Supplementary Fig. S1b,c). Although the optimized conditions led to a lower yield of cross-linked oligomers (Fig. 1b), they were used throughout our study since they ensured chemically well-defined CL Aβ oligomers.

### Aβ42 pentamers and hexamers are artifacts of SDS-PAGE analysis

**Figure 2:**
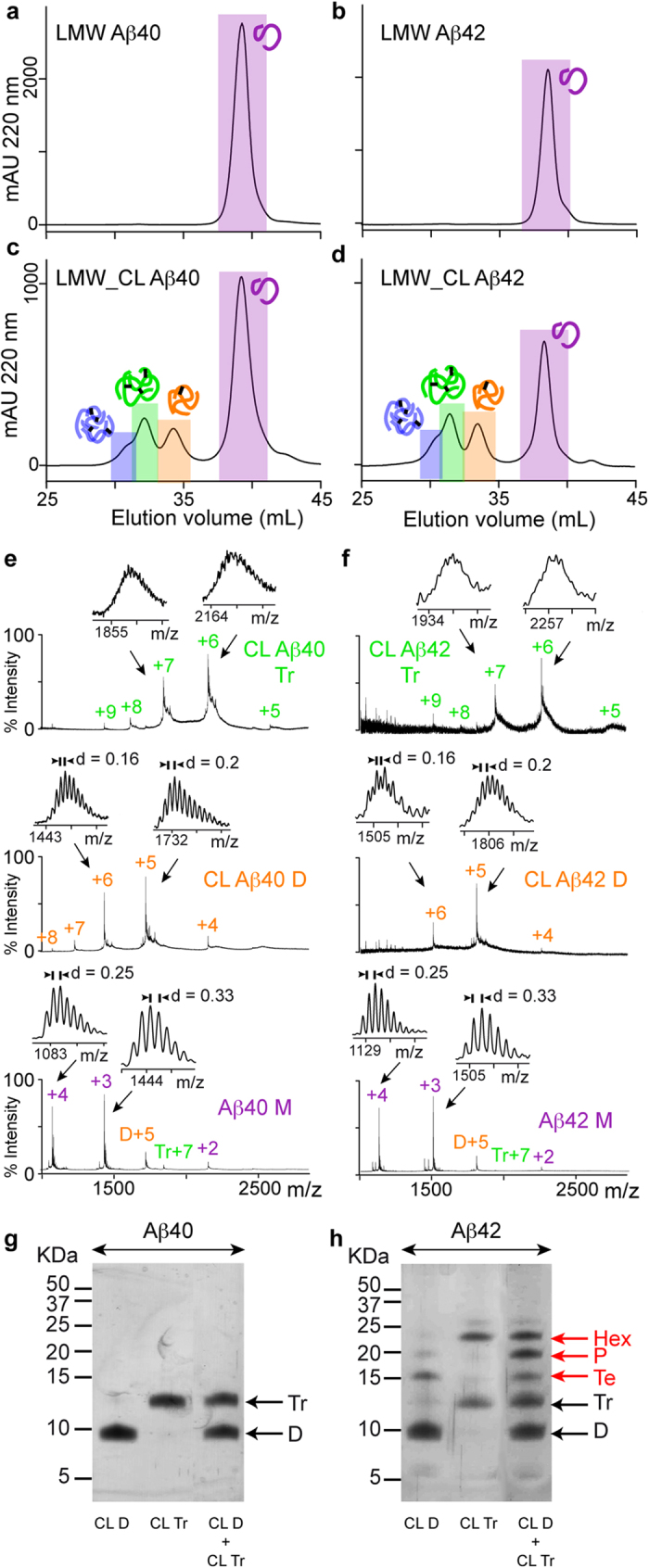
Aβ42 pentamers and hexamers are artifacts of SDS-PAGE analysis. GdnHSCN-SEC analysis of (**a**) LMW Aβ40, (**b**) LMW Aβ42, (**c**) LMW_CL Aβ40, and (**d**) LMW_CL Aβ42. ESI-MS spectra corresponding to peaks detected after GdnHSCN-SEC analysis of (**e**) LMW_CL Aβ40 and (**f**) LMW_CL Aβ42 samples. SDS-PAGE analysis of isolated CL dimers and trimers, as well as mixtures of them for (**g**) Aβ40 and (**h**) Aβ42 obtained after GdnHSCN-SEC fractionation. M = monomers, D = dimers, Tr = trimers, Te = tetramers, P = pentamers, and Hx = hexamers. The red arrows indicate oligomers formed artifactually in the presence of SDS.

Various studies have suggested that SDS affects the oligomerization state of Aβ samples^8^,^19^,^20^. To address this possibility, we sought to determine the oligomer order and distribution of LMW_CL Aβ samples using a new strategy that involved the same principles as those used in SDS-PAGE, that is, denaturation/disaggregation followed by size fractionation, but without using SDS. Denaturation/disaggregation was accomplished by lyophilizing LMW and LMW_CL Aβ40 and Aβ42 samples and later resuspending them in 6.8 M guanidine thiocyanante (GdnHSCN), conditions used to solubilize plaque cores^34^. Therefore, treatment of LMW and LMW_CL Aβ samples with 6.8 M GdnHSCN should break all non-covalent Aβ-Aβ interactions preserving only the covalent ones formed during the cross-linking reaction. Size fractionation of the resulting oligomers was attained by SEC using 10 mM ammonium acetate at pH 8.5 as the elution buffer. This buffer was chosen because it prevented aggregation of the samples during SEC fractionation. After this treatment, referred to as GdnHSCN-SEC analysis, LMW Aβ40 and Aβ42 eluted as a single peak (Fig. 2a,b), confirming that GdnHSCN broke all the non-covalent interactions and that aggregation was prevented during SEC fractionation. In contrast, LMW_CL Aβ40 and Aβ42 samples eluted as four main peaks (Fig. 2c,d). ESI-MS analysis confirmed that the peaks eluting at 39.5, 34.7, and 32.6 mL in LMW_CL Aβ40 and at 38.4, 33.5, and 31.6 mL in LMW_CL Aβ42 samples corresponded to “monomers”, CL dimers, and CL trimers of Aβ40 (Fig. 2e) and Aβ42 (Fig. 2f), respectively. Although peaks eluting at 31.3 mL in LMW_CL Aβ40 (Fig. 2c) and at 30.5 mL in LMW_CL Aβ42 (Fig. 2d) samples were low in abundance, ESI-MS analysis revealed the presence of +8 and +7 charge states corresponding to tetramers. Of note, ESI-MS analysis of peaks corresponding to “monomers” in LMW and LMW_CL Aβ samples also showed that they contained charge states corresponding to non-covalent dimers and trimers (Fig. 2e,f). These observations are consistent with previous results indicating that Aβ monomers exist in rapid equilibrium with low order Aβ oligomers (Fig. 1a)^31^. Altogether, GdnHSCN-SEC analyses indicated that the oligomer distribution for LMW_CL Aβ40 and Aβ42 samples was the same, comprising dimers, trimers and tetramers (Fig. 2c,d). This result is inconsistent with that obtained when the same samples were analyzed by SDS-PAGE (Fig. 1b), thus suggesting that SDS affects Aβ oligomerization, particularly that of Aβ42.

Having access to pure synthetic CL Aβ oligomer samples of well-defined order offered us a unique opportunity to study the effect of SDS on Aβ oligomerization. We analyzed isolated CL Aβ40 and Aβ42 dimers and trimers as well as mixtures of them, obtained after GdnHSCN-SEC, by SDS-PAGE. CL Aβ40 dimers and trimers ran as expected (Fig. 2g). However, CL Aβ42 dimers run also as tetramers, CL Aβ42 trimers also as hexamers, and mixtures of CL Aβ42 dimers and trimers also as tetramers, pentamers and hexamers (Fig. 2h). These findings have two important implications: i) SDS is responsible for affecting the oligomerization state of Aβ42 oligomers. In fact, the temperature and incubation time prior to SDS-PAGE analysis did not have an effect in the oligomerization state of the samples (Supplementary Fig. S2). Consequently, SDS-PAGE is not a reliable technique to characterize the order and distribution of Aβ oligomers present in a sample; ii) they challenge the widely accepted view that pentamers and hexamers are the basic building blocks for Aβ aggregation.

### ESI-IM-MS analysis reveals that Aβ40 and Aβ42 predominantly oligomerize through dimers and trimers

**Figure 3:**
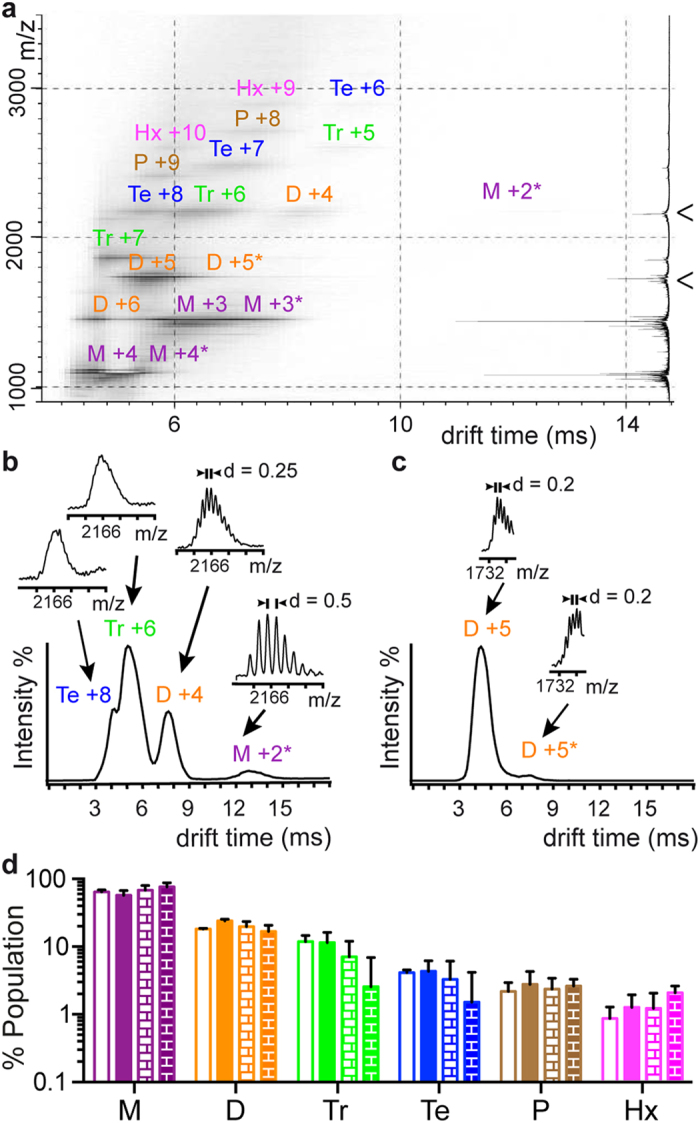
ESI-IM-MS analysis reveals that Aβ40 and Aβ42 oligomerize predominantly through dimers and trimers. (**a**) ESI-IM-MS spectra for LMW Aβ40. M = monomers, D = dimers, Tr = trimers, Te = tetramers, P = pentamers, and Hx = hexamers. The number adjacent to each aggregation state refers to the charge state of the ion. The summed m/z spectrum is shown on the right. Projections of the ESI-IM-MS spectra on the drift time axis for (**b**) m/z 2166 (M +2, D +4, Tr +6, and Te +8) and (**c**) m/z 1732 (D +5), both indicated by an arrowhead in (**a**). For each of the mobility peaks detected, their associated m/z spectrum is also shown. Peaks were assigned following the considerations described in the Supplementary Text. ESI-IM-MS analysis of LMW_CL Aβ40, LMW Aβ42, and LMW_CL Aβ42 samples is shown in Supplementary Figs S3–S5, respectively. Relative population of M (purple), D (orange), Tr (green), Te (blue), P (brown), and Hx (pink) in LMW Aβ40 (empty bars), LMW Aβ42 (filled bars), LMW_CL Aβ40 (empty brick pattern bars) and LMW_CL Aβ42 (filled bricked pattern bars) samples obtained from (**d**) the intensity counts of all charge states detected in ESI-IM-MS spectra.

Given that SDS-PAGE analysis leads to artifacts in the characterization of Aβ oligomers, we consider it critical to establish techniques that can provide reliable results. As detailed in the introduction, ESI-IM-MS is an emerging technique with great potential to characterize the oligomers present in a sample^7^,^10^,^22^, circumventing the problems associated with the use of SDS^8^,^19^,^20^. To evaluate the feasibility of ESI-IM-MS for the characterization of Aβ oligomers, we next studied LMW and LMW_CL Aβ40 and Aβ42 samples in 10 mM ammonium acetate at pH 8.5 (Fig. 3 and Supplementary Figs S3–S5). We began ESI-IM-MS analysis by first assigning all the peaks in each spectrum to specific Aβ oligomers (see Supplementary Text). Briefly, assignments were performed on the basis of the ^13^C isotope distribution (Fig. 3b,c and Supplementary Figs S3b,c–S5b,c), as proposed in the literature^10^. Whenever resolution was not enough, assignment was carried out by the detection of at least two consecutive charge states for a specific Aβ oligomer (Fig. 3a and Supplementary Figs S3a–S5a). The LMW and LMW_CL Aβ40 and Aβ42 samples ranged predominantly from monomers to trimers, although low intensity charge states for tetramers, pentamers and hexamers were also detected (M = monomers, D = dimers, Tr = trimers, Te = tetramers, P = pentamers, and Hx = hexamers) (Fig. 3a and Supplementary Figs S3a–S5a). Next, we determined the oligomer distribution in each sample by using all charge state ions across the whole spectrum (Fig. 3d). There were no significant differences between the oligomer distribution of Aβ40 and Aβ42 when compared in the LMW or LMW_CL samples. Hence, we concluded that the oligomer distribution for Aβ40 and Aβ42 was the same, comprising mainly dimers and trimers, and that PICUP provided an accurate snapshot of this distribution. Noticeably, the ESI-IM-MS oligomer distribution was consistent with the GdnHSCN-SEC analysis (Supplementary Fig. S6), thereby supporting the reliability of ESI-IM-MS for the characterization of Aβ oligomers. Furthermore, in contrast to GdnHSCN-SEC analysis, ESI-IM-MS allowed detection of low abundant oligomers such as pentamers and hexamers and characterization of the oligomer order and distribution of non-covalently linked oligomers.

### Aβ40 and Aβ42 dimers and trimers adopt a globular shape devoid of defined secondary structure

In addition to characterizing the Aβ oligomer order and distribution, ESI-IM-MS has the potential to identify chemical modifications, as well as to provide dynamic and structural information on the oligomers present in the sample. As per the characterization of chemical modifications, ESI-IM-MS analysis revealed that the mass of LMW_CL Aβ oligomers is consistent with the expected loss of two hydrogens for each covalent bond formed in the PICUP reaction (Supplementary Fig. S7)^35^.

To establish the effect of cross-linking on Aβ oligomer dynamic, we measured the line width of the mobility peaks associated with specific Aβ oligomers. The line width corresponding to non-covalent Aβ oligomers in LMW samples was significantly larger than that of CL ones in LMW_CL samples (Supplementary Fig. S8). This finding indicated that during the mobility experiment each of the detected non-covalent Aβ oligomer sampled more conformations than their corresponding CL counterparts. A result consistent with the general expectation that cross-linking, which entails the formation of covalent bonds, reduces the number of accessible conformations.

**Figure 4:**
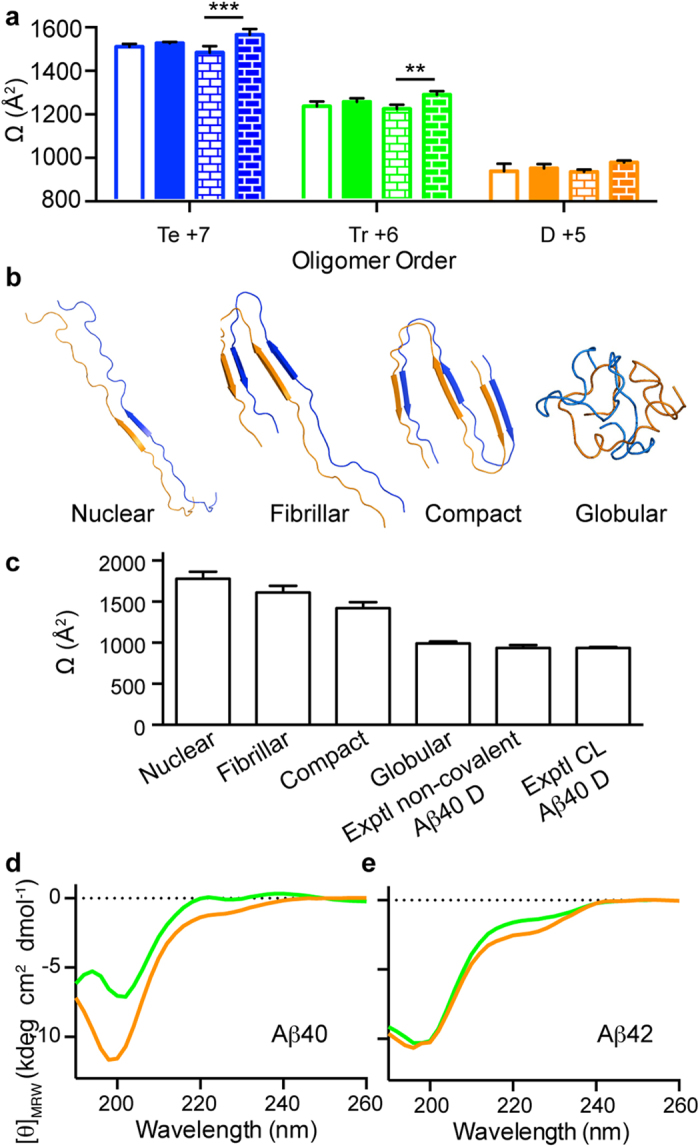
Aβ40 and Aβ42 dimers and trimers adopt a globular shape devoid of defined secondary structure. (**a**) Ω values obtained from the mobility peaks associated with the most abundant charge states for the most abundant Aβ oligomers detected (Te = tetramers, Tr = trimers, and D = dimers) Te +7 (blue), Tr +6 (green), and D +5 (orange) for LMW Aβ40 (empty bars), LMW Aβ42 (filled bars), LMW_CL Aβ40 (empty brick-pattern bars) and LMW_CL Aβ42 (filled brick-pattern bars). Data are the mean ± s.d. of three independent experiments. p-values are calculated using unpaired two-tailed Student’s t-test (**p < 0.01 and ***p < 0.001). (**b**) Theoretical Aβ40 dimer models constructed using reported structural restraints for Aβ aggregates (nuclear^36^, fibrillar^37^, and compact^9^,^38^), as well as those obtained from REMD simulations (globular). (**c**) Comparison of Ω values obtained from theoretical models of the dimer structures described in (b) with the experimental measures for the non-covalent and CL Aβ40 dimers reported in (a). CD analysis of CL dimers (orange) and CL trimers (green) for (**d**) Aβ40 and (**e**) Aβ42 obtained after GdnHSCN-SEC fractionation.

Regarding structural information, ESI-IM-MS experiments can provide collisional cross-section (Ω) values^8^,^11^,^22^,^23^,^24^, which are key structural constraints related to oligomer size and shape. We determined Ω for the most abundant Aβ oligomers detected in the LMW and LMW_CL Aβ40 and Aβ42 samples (Fig. 4a and Supplementary Table 2) and compared the same type of oligomer in the four samples. First, we compared the same type of oligomer in the LMW Aβ40 and Aβ42 samples and no significant differences in drift time (Supplementary Fig. S8a) or Ω values (Fig. 4a) were found, thereby indicating that they were similar. Next, we carried out, the same comparison for the LMW_CL Aβ40 and Aβ42 samples and found significant differences in drift time (Supplementary Fig. S8a) and Ω values (Fig. 4a). This result was not surprising since cross-linking reduces the conformational dynamics of Aβ oligomers (Supplementary Fig. S8) so we expected that differences in shape would be easier to distinguish among the CL oligomers than among the non-covalent ones. However, although this finding might indicate that LMW_CL Aβ42 oligomers have a more extended structure than their Aβ40 counterparts, the difference observed was consistent with the fact that Aβ42 has two more amino acids than Aβ40 (Supplementary Fig. S9). Accordingly, we concluded that Aβ40 and Aβ42 oligomers of the same order adopt the same shape, regardless of whether they are cross-linked.

To rationalize the experimentally obtained Ω values, we constructed three structural models of Aβ dimers for Aβ40 (Fig. 4b) and Aβ42 (Supplementary Fig. S10a). These models were built and named nuclear^36^, fibrillar^37^, or compact^9^,^38^, on the basis of various structural restraints reported for Aβ aggregates. All the predicted Ω values (Fig. 4c and Supplementary Fig. S10b) were markedly larger than the experimental ones, thereby indicating that none of the proposed models fitted the experimental data. Thus, to build more realistic structural models and achieve better sampling, we ran atomistic molecular dynamics (MD) simulations using the replica exchange method (REMD) and simulation conditions similar to those in a ESI-MS experiment^39^,^40^. In the first part of the simulations, all the models, irrespective of their original conformation, collapsed to adopt globular structures (Supplementary Fig. S11). Of the three structural models, the ensemble of globular structures for the nuclear form had the lowest energy (Supplementary Fig. S12). Thus, we took this low-energy ensemble to represent the structure of a globular dimer model (Fig. 4b and Supplementary Fig. S10a). The average Ω value of this ensemble was very similar to the experimental values obtained for the non-covalent and CL dimers (Fig. 4c and Supplementary Fig. S10b), thereby indicating that Aβ dimers have a globular shape. Next, to validate the lack of defined secondary structure adopted by the Aβ oligomers, we measured a CD spectrum of Aβ40 and Aβ42 CL dimers and trimers obtained immediately after SEC (Fig. 4d,e). The spectra for all four samples showed a minimum at around 200 nm, indicative of a lack of defined secondary structure. Additionally, an arm around 230 nm was also observed, which may reflect the existence of dynamic structural nuclei. In summary, the results from ESI-IM-MS, MD simulations, and CD spectroscopy were consistent with Aβ dimers and trimers adopting a globular shape devoid of defined secondary structure (Fig. 4 and Supplementary Fig. S10).

## Discussion

**Figure 5:**
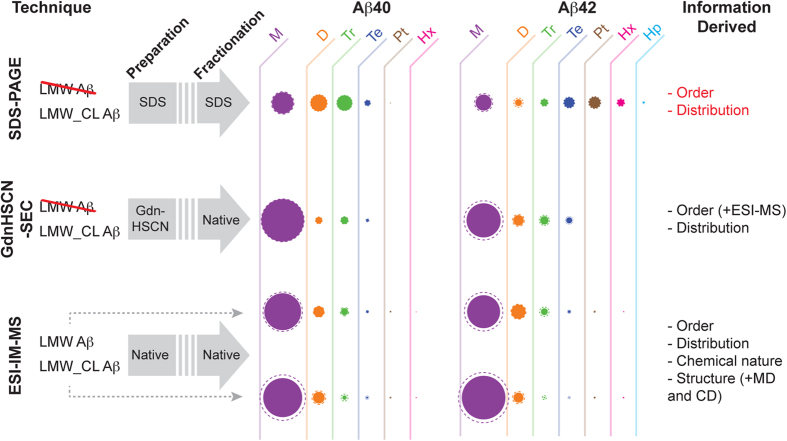
Schematics describing the conditions and the information derived from the different techniques used in this study to characterize LMW and LMW_CL Aβ samples. Techniques involving denaturing conditions in the preparation and/or fractionation of the samples are not suitable for the characterization of LMW Aβ samples. Filled and dashed circles indicate, respectively, the average and the standard deviation of the M = monomers (purple), D = dimers (orange), Tr = trimers (green), Te = tetramers (blue), P = pentamers (brown), Hx = hexamers (pink) and Hp = heptamers (cyan) relative population in each of the samples studied. The information obtained from each of the techniques is shown in the last column. Additional methods used to further support and/or validate specific information derived from either GdnHSCN-SEC or ESI-IM-MS are indicated in parentheses. The results derived from SDS-PAGE analysis are indicated in red because we have shown that this technique provides flawed information regarding the order and distribution of oligomers present in a sample (Fig. 2).

Our results establish that SDS-PAGE leads to artifacts in the determination of the order and distribution of Aβ42 oligomer samples and present ESI-IM-MS as a powerful technique to characterize the order, distribution, chemical modifications, and structure of both covalent and non-covalently linked Aβ oligomers (Fig. 5). Moreover, our findings indicate that pentamers and hexamers are artifacts of SDS-PAGE analysis and reveal Aβ40 and Aβ42 dimers and trimers, adopting a globular structure and lacking defined secondary structure, as the earliest oligomers formed during Aβ aggregation.

Although no other laboratory has studied LMW_CL Aβ40 and Aβ42 samples by ESI-IM-MS, they have addressed LMW Aβ40 and Aβ42 samples^7^,^10^. In this regard, it is important to note that our results are consistent only with studies that used the ^13^C isotopic distribution to assign peaks separated in the mobility dimension. This is the case for findings on LMW Aβ40 samples reported by Dadlez and co-workers^10^. They reported the presence of various oligomers, ranging from dimers to hexadecamers, adopting two conformations, namely compact and extended^13^. The Ω values for the oligomers detected in our study are in agreement with those reported by Dadlez´s group. Differences in the number of oligomers detected in the two studies can be reconciled by taking into account that Dadlez´s work was performed using a higher Aβ40 concentration (200 μM versus 30 μM). On the contrary, our results are not consistent with those of Bowers and co-workers^7^, who detected a single charge state (D -5, they work on negative mode) specific for an Aβ oligomer, which included various mobility peaks, assigned by the authors based upon injection energy studies and PICUP/SDS-PAGE experiments^7^,^21^, to dimers and tetramers for Aβ40, and to dimers, tetramers, hexamers, and dodecamers for Aβ42. These same assignments were further repeated in subsequent papers from the group^41^,^42^,^43^,^44^,^45^. Since their instrumentation did not allow measuring the ^13^C isotope distributions, they could not distinguish whether a mobility peak originated from a structural variant of an oligomer of the same order (*e.g.*, compact and extended forms) or from oligomers of distinct order. We indeed observed two mobility peaks associated with the D+5 charge state for Aβ40 and Aβ42 (Supplementary Fig. S13). However, on the basis of ^13^C isotopic distribution, we assigned these peaks to compact and extended conformations of the dimer rather than to distinct oligomer forms. Therefore, an important conclusion for the application of ESI-IM-MS to the study of Aβ oligomers is that assignments should be based on the ^13^C isotopic distribution of the peaks separated in the mobility dimension, as initially proposed by Dadlez and co-workers^10^. Whenever there is not enough resolution to determine the charge state, we propose that the detection of at least two consecutive charge states for a particular oligomer should be required to assign a mobility peak to a specific oligomer.

A key aspect of the present study is that the GdnHSCN-SEC strategy allowed us access to synthetic Aβ oligomers of well-defined order. This protocol greatly improves previous efforts to fractionate CL Aβ oligomers from SDS-PAGE bands. Indeed, a protocol for the isolation of CL Aβ oligomers of defined order from SDS-PAGE has been described for Aβ40^16^. Attempts to apply the same protocol to CL Aβ42 samples were unsuccessful^16^. According to the authors of that study, isolated CL Aβ42 oligomers were not stable upon re-electrophoreses. This result reinforces our findings that Aβ42 pentamers and hexamers are not *bona fide* cross-linked oligomers but are dimers and trimers that evolve into pentamers and hexamers in the presence of SDS. Having developed a fractionation protocol that permitted obtaining Aβ oligomers of well-defined order also allowed us to characterize them by CD spectroscopy. By combining CD analysis with structural information derived from Ω values obtained from ESI-IM-MS studies, and atomistic MD simulations, we show that dimers and trimers have a globular fold lacking defined secondary structure (Fig. 4 and Supplementary Fig. S10). This observation contrasts with previous publications where SDS-PAGE-purified CL Aβ40 dimers, trimers, and tetramers were suggested to adopt a β-sheet structure^16^. In the aforementioned work, the authors used PICUP conditions that, in our hands, afforded a heterogeneous mixture of oxidized products when analyzed by RP-HPLC and LC-HRMS (Supplementary Fig. S1 and Table S1). Moreover, the authors fractionated the oligomers using SDS-PAGE, and although several purification steps were applied, there is the possibility that SDS contaminated the samples, thus affecting the structure of the purified oligomers.

In summary, our results reveal that SDS-PAGE leads to artifactual results in determining the order and distribution of Aβ42 oligomers, while ESI-IM-MS is established as a powerful technique to characterize the order, distribution, chemical modifications, and structure of both covalent and non-covalently linked Aβ40 and Aβ42 oligomers. These findings challenge previous scientific paradigms in the AD field built upon the use of SDS-PAGE to characterize Aβ oligomers samples. Among these paradigms, pentamers and hexamers have been established as the building blocks for Aβ aggregation. However, here we demonstrate that pentamers and hexamers form artifactually from dimers and trimers in the presence of SDS. Importantly, we identify Aβ40 and Aβ42 dimers and trimers, adopting a globular structure and lacking defined secondary structure, as the earliest oligomers formed during Aβ aggregation. This result is in agreement with an elegant study addressing homo- and hetero-molecular Aβ40/Aβ42 aggregation^46^. This study reveals that from all possible stages of aggregation, Aβ40 and Aβ42 interact significantly only at the level of primary nucleation. The authors explain this result by suggesting that the structure of primary nuclei is less organized than that of the fibrils. This lower level of organization would allow for better accommodation of the two-residue C-terminal mismatch. Our findings indicating that Aβ40 and Aβ42 dimers and trimers adopt very similar shapes lacking defined secondary structure would be in complete agreement with the proposed explanation. Finally, since structural models for other higher order Aβ oligomers have been reported to adopt a β-sheet structure^9^,^38^,^47^, our findings that Aβ dimers and trimers are globular and lack defined secondary structure indicate that major structural rearrangements occur during fibril formation. These observations highlight the importance of defining the structures and properties of the various species involved in Aβ aggregation. Indeed, it is only through this level of molecular detail that we will be able to interfere with Aβ aggregation in a rational manner.

## Methods

### Preparation of LMW Aβ40 and Aβ42

Aβ peptides of 40 and 42 residues, Aβ40 and Aβ42, were synthesized and purified by Dr. James I. Elliott (New Haven). LMW Aβ40 and Aβ42 preparations were obtained using Size-Exclusion Chromatography (SEC). Aβ peptide was dissolved in 6.8 M GdnHSCN (Life Technologies) at 8.5 mg/mL, sonicated for 5 min, and diluted to 5 mg/mL of peptide and 4 M GdnHSCN with H_2_O. It was then centrifuged at 10,000 *g* for 6 min at 4 °C and passed through a 0.45-μm Millex filter (Millipore). The resulting Aβ solution was injected into a HiLoad Superdex 75 prep grade column (GE Healthcare). The column was equilibrated using 10 mM sodium phosphate pH 7.4 and eluted at 4 °C at a flow rate of 1 mL/min. The peak attributed to LMW Aβ was collected and its protein concentration determined (see section Quantifying Aβ40 and Aβ42 protein concentration). The peptide solution was then diluted to 150 μM, frozen, and kept at −20 °C until used.

### Quantifying Aβ40 and Aβ42 protein concentration

LMW Aβ concentration obtained from SEC fractions was determined by reversed phase high-performance liquid chromatography (RP-HPLC) coupled to photodiode array detector (PDA) (Waters Alliance 2695 equipped with 2998 photodiode array detector). RP-HPLC analysis was carried out using a Symmetry 300 C_4_ column (4.6 × 150 mm, 5 μm, 300 Å; Waters), a flow rate of 1 mL/min, and a linear gradient from 0 to 60% B in 15 min (A = 0.045% trifluoroacetic acid (TFA) in water, and B = 0.036% TFA in acetonitrile) at 60 °C. A calibration curve was generated based on Aβ40 and Aβ42 solutions that had previously been quantified by amino acid analysis.

### Photo-Induced Cross-linking of Unmodified Proteins (PICUP)

The PICUP reaction was initially run following descriptions in the literature^33^. The experimental set-up consisted of a camera body and a 150-W slide projector. A PCR tube containing the reaction mixture to be cross-linked was placed inside the camera body for irradiation. The sample was irradiated via the slide projector for a short time, precisely controlled by the camera shutter. PICUP reactions were done using an Aβ/Ru(bpy)_3_^2+^/APS ratio of 1:2:40. To this end, 4 μL of 3 mM Ru(bpy)_3_^2+^ and 4 μL of 60 mM APS were added to 40 μL of 150 μM LMW Aβ in 10 mM sodium phosphate buffer. The mixture was irradiated for 1 s at a distance of 10 cm and immediately quenched by adding 6.5 μL of 4 M dithiothreitol (DTT). Later on, reaction conditions were optimized to prevent Aβ oxidation (Supplementary Fig. S1). The best conditions were found when using a lower proportion of APS, Aβ/Ru(bpy)_3_^2+^/APS 1:2:5, a distance of 10 cm, and an irradiation time of 1 s. Unless otherwise stated, we used an Aβ/Ru(bpy)_3_^2+^/APS ratio of 1:2:5 to prevent formation of oxidized byproducts and to obtain chemically well-defined cross-linked oligomers. After the PICUP reaction, the reagents, which are incompatible with ESI-IM-MS and RP-HPLC analysis, were removed using Bio-Spin P30 columns (Bio-Rad) equilibrated in 10 mM ammonium acetate at pH 8.5. For comparative purposes, PICUP samples were also passed through Bio-Spin P30 columns when analyzed by SDS-PAGE.

### SDS-PAGE

10 μL of 3X sample buffer (SB) (150 μl 10% SDS, 75 μl 4 M DTT, 400 μl H_2_O, 375 μl 8X sample buffer −8 mL 1 M Tris pH 6.8, 9.3 mL 87% Glycerol, 5 mg Coomassie Brilliant Blue G, 2.8 mL H_2_O) was added to 20 μL of the sample to be analyzed by SDS-PAGE. The samples were boiled at 95 °C for 5 min, unless otherwise stated, and kept at −20 °C until used. A 20-μL aliquot of each sample was electrophoresed in 0.75 mm-thick SDS-PAGE gels containing 15% acrylamide. Gels were run at 80–100 V and silver-stained.

### RP-HPLC

RP-HPLC analysis of LMW_CL Aβ40 samples was done using a Symmetry 300 C_4_ column (4.6 × 150 mm, 5 μm, 300 Å; Waters) at a flow rate of 1 mL/min and a linear gradient from 0 to 60% B in 15 min (A = 0.045% TFA in water, and B = 0.036% TFA in acetonitrile) at 60 °C.

***LC-HRMS.*** 

180 pmols of LMW_CL Aβ40 and LMW_CL Aβ42 (Supplementary Fig. S14) were analyzed using a BioSuite pPhenyl 1000 analytical column (10 μm, 2 × 75 mm; Waters) at a flow rate of 100 μl/min comprising a linear gradient running from 5 to 80% B in 60 min (A = 0.1% formic acid (FA) in water, B = 0.1% FA in acetonitrile). The column outlet was directly connected to an Advion TriVersa NanoMate, which was used as a splitter and as the nanospray source of an LTQ-FT Ultra mass spectrometer (Thermo Scientific). Positive polarity was used with a spray voltage in the NanoMate source set to 1.7 kV. The capillary voltage, capillary temperature, and tube lens on the LTQ-FT were tuned to 40 V, 200 °C, and 100 V, respectively.

### GdnHSCN-SEC

Although methionine oxidation was minimized by using Aβ/Ru(bpy)_3_^2+^/APS ratios of 1:2:5, a small percentage of oxidized methionine was detected after isolation of CL Aβ dimers and trimers. To completely reduce all oxidized methionine side-chains, 500 μL of the quenched PICUP reaction were lyophilized and resuspended in TFA containing 30 equivalents of Me_2_S-NH_4_I and left for 2 hrs at 4 °C on a rotating wheel. Afterwards, TFA was evaporated under a stream of N_2_ and 500 μL of H_2_O was added to the sample. The mixture was then lyophilized, resuspended in 6.8 M GdnHSCN at an Aβ concentration of 8.5 mg/mL, and subsequently diluted with H_2_O to 5 mg/mL of peptide and 4M GdnHSCN. The resulting Aβ solution was injected into three columns in series, Superdex 75 HR 10/300-Superdex 75 HR 10/300-Superdex 200 HR10/300. The columns were equilibrated in 10 mM ammonium acetate pH 8.5, and the samples eluted at 4 °C at a flow rate of 0.5 mL/min.

### ESI-IM-MS

ESI-IM-MS experiments were performed on a Synapt HDMS (Waters) quadrupole-traveling wave IMS-oaTOF mass spectrometer equipped with an Advion TriVersa NanoMate (Advion Biosciences). Positive ESI was used with a capillary voltage of 1.7 kV. A sampling cone voltage of 40 V and a backing pressure of 5.7 mbar were set for the observation of low-n Aβ oligomers. Although no signal was detected over 3000 m/z, data were acquired over the m/z range of 500 to 5000 for 2 min. The ion accelerating voltage in the trap T-wave device was 6 V unless otherwise stated; the ion accelerating voltage in the transfer T-wave device was kept constant at 4 V. The IM gas flow was kept at 23 mL/min. ESI-IM-MS data were obtained at three wave heights: 7, 7.5 and 8. The drift time Ω function was calibrated using denatured ubiquitin (charge states +9 to +11), myoglobin (charge states +15 to +22) and cytochrome C (charge states +11 to +18). Drift times were corrected for both mass-dependent and mass-independent times^48^,^49^. All unknown drift time values, for which a Ω was derived, fell within the calibration curve obtained using the 19 charge states of the three protein standards. The raw data were processed using Mass Lynx v4.1 software (Waters). The reported data are the average of three independent experiments. The considerations to assign ESI-IM-MS peaks to specific Aβ oligomers are described in Supplementary text. To determine the low-n Aβ oligomer distribution for all four samples under study, we used all charge state ions across the whole spectra. For those charge states that contained contributions from different Aβ species in the mobility dimension, the relative contribution of each one was determined by fitting the mobilogram to a sum of Gaussian functions *g*(x_c_, w, A), where x_c_, w, and A correspond to the center, the width at half-height, and the area of the Gaussian peak, respectively. The fitted function obtained for each mobilogram, f_im, can be written as:


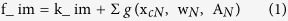


where k_im is the baseline intensity of the mobilogram, Σ *g*(x_c*N*_, w_*N*_, A_*N*_) is the sum of Gaussians contributing to each mobilogram, and *N* is the number of Gaussian/species used to fit each mobilogram. The relative population of a species contributing to a given charge state was obtained by dividing the area of the peak representing that species by the sum of the areas of each of the other species contributing to the given charge state. The contribution of a species to the overall intensity of a charge state ion was obtained by multiplying it by its relative population. The population of a species was obtained by summing the intensity of all charge states specific for the particular species and dividing it by the sum of the intensities of all charge state ions across the whole spectra.

### Modeling Aβ dimer structure

#### Construction of Aβ dimer models

Three structural models for Aβ40 and Aβ42 dimers were built, taking into account reported structural restraints for Aβ aggregates (nuclear^36^, fibrillar^37^, and compact^9^,^38^) (Fig. 4b for Aβ40, Supplementary Fig. S10a for Aβ42). The sequence of Aβ was divided into three segments: the N-terminus (residues 1 to 11), the middle segment (residues 12 to 26), and the C-terminus (residues 27 to 40 for Aβ40, or 27 to 42 for Aβ42). In all the models, the two Aβ units interact through β-strand regions defined within each Aβ unit to form parallel β-sheets through intermolecular hydrogen bonds. Previous reports have proposed that the region comprising residues 17 to 21 nucleates Aβ self-assembly^36^. In the nuclear model, residues 12 to 24 adopt a β-strand conformation. In the fibrillar model, the regions spanning residues 12 to 24 and residues 30 to 40 (for Aβ40) or 42 (for Aβ42) adopt a β-strand conformation. Residues 25–29 contain a bend that brings the two β-strands into contact through side chain-side chain interactions. This model corresponds to the structure of Aβ when it is incorporated into the fibril^37^. The compact model was constructed taking into account structural models of specific Aβ oligomer preparations that reveal a stable N-terminal β-strand^9^,^38^. Thus, each Aβ unit contained three β-strand regions comprising residues 1 to 6, 12 to 24, and 30 to 40 (for Aβ40) or 42 (for Aβ42). Residues 7 to 11 and 25 to 29 include a bend of the peptide that brings the three β-strands into contact through side chain-side chain interactions. Each of the six structural models, three for Aβ40 and three for Aβ42, were constructed using PyMol and Modeller tools starting from the Aβ40 fibril structure derived from solid-state Nuclear Magnetic Resonance Spectroscopy (ssNMR) data (Protein Data Bank ID is 2LMN)^37^.

#### Structure equilibration in water

The six structural models were built, optimized, solvated in a truncated octahedron box, and then subsequently neutralized with six sodium ions^50^. Transferable Interaction Potential 3 point (TIP3P)^51^ parameters were used for water and AMBER99SB-ILDN force field^52^ for proteins. Simulations in water were done using periodic boundary conditions and the particle mesh Ewald method^53^ for long-range electrostatic treatment (0.12 nm grid size), combined with a cut-off radius of 1 nm for Lennard-Jones interactions. All aqueous simulations were done at the isothermal-isobaric ensemble (T = 300 K and P = 1 atm) using a Berendsen thermostat and barostat^54^. The SHAKE algorithm^55^ was used to constrain all bond distances to their equilibrium value, allowing a 2-fs integration time step for solution conditions. We performed 5 ns of equilibration in water and computed and minimized the averaged structure of snapshots collected in the last ns. Ω values for each of the structural models were computed with the exact hard sphere scattering method (EHSS), which consists of a trajectory calculation using hard spheres centered at the position of each atom^56^.

#### REMD simulation in the gas phase

As a starting structure for REMD simulation, water molecules and counter ions were removed. We used the REMD method^57^ to perform extended simulations at a range of temperatures for the three models of Aβ40 and Aβ42 dimers in the gas phase. The simulations were done considering a charge state +5, which is the most populated charge state for dimers in the ESI-MS profile. We chose 19 simulation temperatures for each structure, covering a range from 300 K to 614 K, which corresponds to the temperature variability range in the mobility measurements^58^. For gas phase simulations, we did not use any cut-off for non-bonded interactions. We used the SHAKE algorithm for constraining bonds and a 1-fs integration time step. For each Aβ dimer structure, we performed 1 μs of REMD simulation. For the analysis, we considered the trajectories at 300 K, a temperature that simulates ideally mild vaporization conditions, and used the Gromacs.4.5.3 package. Ω values for the ensemble of structures generated during the last 500 ns of REMD, were also computed with the exact hard sphere scattering method (EHSS)^56^.

### Far-UV CD Spectroscopy

CD spectra were recorded on a Jasco 815 spectrometer from 190 to 250 nm with a data pitch of 0.2 nm, a bandwidth of 2 nm, and a scan speed of 50 nm/min with a 4-s response time. A 1-cm cell was used. After GdnHSCN-SEC fractionation using three columns in series equilibrated in 10 mM sodium phosphate at pH 8.5, spectra for CL Aβ40 and Aβ42 dimers, and trimers were acquired at 4 °C. CD data were analyzed using the SpectraManager program.

### Summary of statistical analysis

GraphPad Prism was used for all statistical analyses. The data are presented as mean ± s.d. except when stated otherwise. The Student’s t-test (unpaired, two-tailed) was used to calculate statistical significance. The images shown are representative of those obtained in at least three independent experiments.

## Additional Information

**How to cite this article**: Pujol-Pina, R. *et al.* SDS-PAGE analysis of Aβ oligomers is disserving research into Alzheimer’s disease: appealing for ESI-IM-MS. *Sci. Rep.* **5**, 14809; doi: 10.1038/srep14809 (2015).

## Supplementary Material

Supplementary Information

## References

[b1] Haass C. & Selkoe D. J. Soluble protein oligomers in neurodegeneration: lessons from the Alzheimer’s amyloid beta-peptide. Nat. Rev. Mol. Cell Biol. 8, 101–112 (2007). 17245412 10.1038/nrm2101

[b2] Hefti F. *et al.* The case for soluble Abeta oligomers as a drug target in Alzheimer’s disease. Trends Pharmacol. Sci. 34, 261–266 (2013). 23582316 10.1016/j.tips.2013.03.002

[b3] Editorial. State of aggregation. Nat. Neurosci. 14, 399 (2011). 21445061 10.1038/nn0411-399

[b4] Benilova I., Karran E. & De Strooper B. The toxic Abeta oligomer and Alzheimer’s disease: an emperor in need of clothes. Nat. Neurosci. 15, 349–357 (2012). 22286176 10.1038/nn.3028

[b5] Bitan G. *et al.* Amyloid beta-protein (Abeta) assembly: Abeta 40 and Abeta 42 oligomerize through distinct pathways. Proc. Natl. Acad. Sci. USA 100, 330–335 (2003). 12506200 10.1073/pnas.222681699 PMC140968

[b6] Necula M., Kayed R., Milton S. & Glabe C. G. Small molecule inhibitors of aggregation indicate that amyloid beta oligomerization and fibrillization pathways are independent and distinct. J. Biol. Chem. 282, 10311–10324 (2007). 17284452 10.1074/jbc.M608207200

[b7] Bernstein S. L. *et al.* Amyloid-beta protein oligomerization and the importance of tetramers and dodecamers in the aetiology of Alzheimer’s disease. Nat. Chem. 1, 326–331 (2009). 20703363 10.1038/nchem.247 PMC2918915

[b8] Yu L. *et al.* Structural characterization of a soluble amyloid beta-peptide oligomer. Biochemistry 48, 1870–1877 (2009). 19216516 10.1021/bi802046n

[b9] Ahmed M. *et al.* Structural conversion of neurotoxic amyloid-beta(1-42) oligomers to fibrils. Nat. Struct. Mol. Biol. 17, 561–567 (2010). 20383142 10.1038/nsmb.1799 PMC2922021

[b10] Kloniecki M. *et al.* Ion mobility separation coupled with MS detects two structural states of Alzheimer’s disease Abeta1-40 peptide oligomers. J. Mol. Biol. 407, 110–124 (2011). 21237171 10.1016/j.jmb.2011.01.012

[b11] Lee J., Culyba E. K., Powers E. T. & Kelly J. W. Amyloid-beta forms fibrils by nucleated conformational conversion of oligomers. Nat. Chem. Biol. 7, 602–609 (2011). 21804535 10.1038/nchembio.624 PMC3158298

[b12] Narayan P. *et al.* The extracellular chaperone clusterin sequesters oligomeric forms of the amyloid-beta(1–40) peptide. Nat. Struct. Mol. Biol. 19, 79–83 (2012). 22179788 10.1038/nsmb.2191 PMC4979993

[b13] Bitan G. & Teplow D. B. Rapid photochemical cross-linking–a new tool for studies of metastable, amyloidogenic protein assemblies. Acc. Chem. Res. 37, 357–364 (2004). 15196045 10.1021/ar000214l

[b14] Citron M. *et al.* Mutant presenilins of Alzheimer’s disease increase production of 42-residue amyloid beta-protein in both transfected cells and transgenic mice. Nat. Med. 3, 67–72 (1997). 8986743 10.1038/nm0197-67

[b15] Blennow K., Zetterberg H. & Fagan A. M. Fluid biomarkers in Alzheimer disease. Cold Spring Harb. Perspect. Med. 2, a006221 (2012). 22951438 10.1101/cshperspect.a006221 PMC3426814

[b16] Ono K., Condron M. M. & Teplow D. B. Structure-neurotoxicity relationships of amyloid beta-protein oligomers. Proc. Natl. Acad. Sci. USA 106, 14745–14750 (2009). 19706468 10.1073/pnas.0905127106 PMC2736424

[b17] Paravastu A. K., Qahwash I., Leapman R. D., Meredith S. C. & Tycko R. Seeded growth of beta-amyloid fibrils from Alzheimer’s brain-derived fibrils produces a distinct fibril structure. Proc. Natl. Acad. Sci. USA 106, 7443–7448 (2009). 19376973 10.1073/pnas.0812033106 PMC2678625

[b18] Roychaudhuri R., Yang M., Hoshi M. M. & Teplow D. B. Amyloid beta-protein assembly and Alzheimer disease. J. Biol. Chem. 284, 4749–4753 (2009). 18845536 10.1074/jbc.R800036200 PMC3837440

[b19] Bitan G., Fradinger E. A., Spring S. M. & Teplow D. B. Neurotoxic protein oligomers–what you see is not always what you get. Amyloid 12, 88–95 (2005). 16011984 10.1080/13506120500106958

[b20] Watt A. D. *et al.* Oligomers, fact or artefact? SDS-PAGE induces dimerization of beta-amyloid in human brain samples. Acta Neuropathol. 125, 549–564 (2013). 23354835 10.1007/s00401-013-1083-z

[b21] Bernstein S. L. *et al.* Amyloid beta-protein: monomer structure and early aggregation states of Abeta42 and its Pro19 alloform. J. Am. Chem. Soc. 127, 2075–2084 (2005). 15713083 10.1021/ja044531p

[b22] Smith D. P., Radford S. E. & Ashcroft A. E. Elongated oligomers in beta2-microglobulin amyloid assembly revealed by ion mobility spectrometry-mass spectrometry. Proc. Natl. Acad. Sci. USA 107, 6794–6798 (2010). 20351246 10.1073/pnas.0913046107 PMC2872402

[b23] Sitkiewicz E., Oledzki J., Poznanski J. & Dadlez M. Di-tyrosine cross-link decreases the collisional cross-section of Abeta peptide dimers and trimers in the gas phase: an ion mobility study. PloS one 9, e100200 (2014). 24945725 10.1371/journal.pone.0100200 PMC4063900

[b24] Sitkiewicz E., Kloniecki M., Poznanski J., Bal W. & Dadlez M. Factors influencing compact-extended structure equilibrium in oligomers of Abeta1-40 peptide–an ion mobility mass spectrometry study. J. Mol. Biol. 426, 2871–2885 (2014). 24857861 10.1016/j.jmb.2014.05.015

[b25] Hernandez H. & Robinson C. V. Determining the stoichiometry and interactions of macromolecular assemblies from mass spectrometry. Nat. Protoc. 2, 715–726 (2007). 17406634 10.1038/nprot.2007.73

[b26] Ayed A., Krutchinsky A. N., Ens W., Standing K. G. & Duckworth H. W. Quantitative evaluation of protein-protein and ligand-protein equilibria of a large allosteric enzyme by electrospray ionization time-of-flight mass spectrometry. Rapid Commun. Mass Spectrom. 12, 339–344 (1998). 9554114 10.1002/(SICI)1097-0231(19980415)12:7<339::AID-RCM163>3.0.CO;2-6

[b27] Gabelica V., Galic N., Rosu F., Houssier C. & De Pauw E. Influence of response factors on determining equilibrium association constants of non-covalent complexes by electrospray ionization mass spectrometry. J. Mass Spectrom. 38, 491–501 (2003). 12794869 10.1002/jms.459

[b28] Chitta R. K., Rempel D. L. & Gross M. L. Determination of affinity constants and response factors of the noncovalent dimer of gramicidin by electrospray ionization mass spectrometry and mathematical modeling. J. Am. Soc. Mass Spectrom. 16, 1031–1038 (2005). 15914025 10.1016/j.jasms.2005.04.001

[b29] Liu J. & Konermann L. Protein-protein binding affinities in solution determined by electrospray mass spectrometry. J. Am. Soc. Mass Spectrom. 22, 408–417 (2011). 21472560 10.1007/s13361-010-0052-1

[b30] Boeri Erba E., Barylyuk K., Yang Y. & Zenobi R. Quantifying protein-protein interactions within noncovalent complexes using electrospray ionization mass spectrometry. Anal. Chem. 83, 9251–9259 (2011). 22047453 10.1021/ac201576e

[b31] Teplow D. B. Preparation of amyloid beta-protein for structural and functional studies. Methods Enzymol. 413, 20–33 (2006). 17046389 10.1016/S0076-6879(06)13002-5

[b32] Jan A., Hartley D. M. & Lashuel H. A. Preparation and characterization of toxic Abeta aggregates for structural and functional studies in Alzheimer’s disease research. Nat. Protoc. 5, 1186–1209 (2010). 20539293 10.1038/nprot.2010.72

[b33] Bitan G. Structural study of metastable amyloidogenic protein oligomers by photo-induced cross-linking of unmodified proteins. Methods Enzymol. 413, 217–236 (2006). 17046399 10.1016/S0076-6879(06)13012-8 PMC2782599

[b34] Selkoe D. J., Abraham C. R., Podlisny M. B. & Duffy L. K. Isolation of low-molecular-weight proteins from amyloid plaque fibers in Alzheimer’s disease. J. Neurochem. 46, 1820–1834 (1986). 3517233 10.1111/j.1471-4159.1986.tb08501.x

[b35] Fancy D. A. & Kodadek T. Chemistry for the analysis of protein-protein interactions: rapid and efficient cross-linking triggered by long wavelength light. Proc. Natl. Acad. Sci. USA 96, 6020–6024 (1999). 10339534 10.1073/pnas.96.11.6020 PMC26828

[b36] Reinke A. A., Ung P. M., Quintero J. J., Carlson H. A. & Gestwicki J. E. Chemical probes that selectively recognize the earliest Abeta oligomers in complex mixtures. J. Am. Chem. Soc. 132, 17655–17657 (2010). 21105683 10.1021/ja106291e PMC3005376

[b37] Petkova A. T., Yau W. M. & Tycko R. Experimental constraints on quaternary structure in Alzheimer’s beta-amyloid fibrils. Biochemistry 45, 498–512 (2006). 16401079 10.1021/bi051952q PMC1435828

[b38] Haupt C. *et al.* Structural basis of beta-amyloid-dependent synaptic dysfunctions. Angew. Chem. Int. Ed. Engl. 51, 1576–1579 (2012). 22234970 10.1002/anie.201105638

[b39] Arcella A. *et al.* Structure and dynamics of oligonucleotides in the gas phase. Angew. Chem. Int. Ed. Engl. 54, 467–471 (2015). 25417598 10.1002/anie.201406910

[b40] Meyer T., Gabelica V., Grubmüller H. & Orozco M. Proteins in the gas phase. WIRES Comput. Mol. Sci. 3, 408–425 (2012).

[b41] Murray M. M. *et al.* Amyloid beta protein: Abeta40 inhibits Abeta42 oligomerization. J. Am. Chem. Soc. 131, 6316–6317 (2009). 19385598 10.1021/ja8092604 PMC2697393

[b42] Gessel M. M., Bernstein S., Kemper M., Teplow D. B. & Bowers M. T. Familial Alzheimer’s disease mutations differentially alter amyloid beta-protein oligomerization. ACS Chem. Neurosci. 3, 909–918 (2012). 23173071 10.1021/cn300050d PMC3503339

[b43] Gessel M. M. *et al.* Abeta(39–42) modulates Abeta oligomerization but not fibril formation. Biochemistry 51, 108–117 (2012). 22129303 10.1021/bi201520b PMC3271797

[b44] Zheng X. *et al.* Z-Phe-Ala-diazomethylketone (PADK) disrupts and remodels early oligomer states of the Alzheimer disease Abeta42 protein. J. Biol. Chem. 287, 6084–6088 (2012). 22253440 10.1074/jbc.C111.328575 PMC3307282

[b45] Zheng X. *et al.* Amyloid beta-protein assembly: The effect of molecular tweezers CLR01 and CLR03. J. Phys Chem. B 119, 4831–4841 (2015). 25751170 10.1021/acs.jpcb.5b00692 PMC4415044

[b46] Cukalevski R. *et al.* The Aβ40 and Aβ42 peptides self-assemble into separate homomolecular fibrils in binary mixtures but cross-react during primary nucleation. Chem. Sci. 6, 4215–4233 (2015). 10.1039/c4sc02517b PMC5707514 29218188

[b47] Lendel C. *et al.* A hexameric peptide barrel as building block of amyloid-beta protofibrils. Angew. Chem. Int. Ed. Engl. 53, 12756–12760 (2014). 25256598 10.1002/anie.201406357

[b48] Ruotolo B. T., Benesch J. L., Sandercock A. M., Hyung S. J. & Robinson C. V. Ion mobility-mass spectrometry analysis of large protein complexes. Nat. Protoc. 3, 1139–1152 (2008). 18600219 10.1038/nprot.2008.78

[b49] Smith D. P. *et al.* Deciphering drift time measurements from travelling wave ion mobility spectrometry-mass spectrometry studies. Eur. J. Mass. Spectrom. 15, 113–130 (2009). 10.1255/ejms.947 19423898

[b50] Smith D. E. & Dang L. X. Computer simulations of NaCl association in polarizable water. J. Chem. Phys. 100, 3757–3766 (1994).

[b51] Jorgensen W. L., Chandrasekhar J., Madura J. D., Impey R. W. & Klein M. L. Comparison of Simple Potential Functions for Simulating Liquid Water. J. Chem. Phys. 79, 926–935 (1983).

[b52] Lindorff-Larsen K. *et al.* Improved side-chain torsion potentials for the Amber ff99SB protein force field. Proteins 78, 1950–1958 (2010). 20408171 10.1002/prot.22711 PMC2970904

[b53] York D. M., Darden T. A. & Pedersen L. G. The effect of long‐range electrostatic interactions in simulations of macromolecular crystals: A comparison of the Ewald and truncated list methods. J. Chem. Phys. 99, 8345–8348 (1993).

[b54] Berendsen H. J. C., Postma J. P. M., van Gunsteren W. F., DiNola A. & Haak J. R. Molecular dynamics with coupling to an external bath. J. Chem. Phys. 81, 3684–3690 (1984).

[b55] Ryckaert P., Cicotti G. & Berendsen H. J. C. Numerical Integration of the Cartesian Equations of Motion of a System with Constraints: Molecular Dynamics of n-Alkanes. J. Comput. Phys. 23, 327–341 (1977).

[b56] Shvartsburg A. A. & Jarrold M. F. An exact hard-spheres scattering model for the mobilities of polyatomic ions. Chem. Phys. Lett. 261, 86–91 (1996).

[b57] Sugita Y. & Okamoto Y. Replica-exchange molecular dynamics method for protein folding. Chem. Phys. Lett. 314, 141–151 (1999).

[b58] Morsa D., Gabelica V. & De Pauw E. Effective temperature of ions in traveling wave ion mobility spectrometry. Anal. Chem. 83, 5775–5782 (2011). 21682304 10.1021/ac201509p

