# Supplementary material for: SDS-PAGE analysis of Aβ oligomers is disserving research into Alzheimer´s disease: appealing for ESI-IM-MS
Source: Sci Rep. 2015 Oct 9;5:14809. doi: 10.1038/srep14809 (PMC4598734; doi:10.1038/srep14809)
Supplement: Supplementary Information [file srep14809-s1.pdf]

Supplementary Information

SDS-PAGE analysis of Aβ oligomers is disserving research into Alzheimer´s disease: appealing for ESI-IM-MS

Rosa Pujol-Pina,[1#] Sílvia Vilaprinyó-Pascual,[1#] Roberta Mazzucato,[1] Annalisa Arcella,[2] Marta Vilaseca,[3] Modesto Orozco,[2,4] Natàlia Carulla[1*]

[1]Institute for Research in Biomedicine (IRB Barcelona), Baldiri Reixac 10, Barcelona 08028, Spain

[2]Joint IRB-BSC Research Program in Computational Biology, Institute for Research in Biomedicine (IRB Barcelona), Baldiri Reixac 10, Barcelona 08028, Spain

[3]Mass Spectrometry Core Facility, Institute for Research in Biomedicine (IRB Barcelona), Baldiri Reixac 10, Barcelona 08028, Spain

[4]Department of Biochemistry and Molecular Biology, University of Barcelona, Diagonal 647, Barcelona 08028, Spain

[#]Both authors have contributed equally to this work

[*]Corresponding author: Natàlia Carulla, Baldiri Reixac 10, Barcelona 08028, Spain, telephone: +34 93 4037123, fax: +34 93 4037126, e-mail: natalia.carulla@irbbarcelona.org

Supplementary Text

Assignment of ESI-IM-MS spectra to specific Aβ oligomers

We assigned the ESI-IM-MS peaks to specific Aβ oligomers on the basis of several considerations.

Firstly, IM separations are proportional to the Ω/z. For any charge state m/z observed in the mass spectrum, several species could be present (e.g. M^{+z}, D^{+2z}, Tr^{+3z}, Te^{+4z}; where M = monomers, D = dimers, Tr = trimers, and Te = tetramers). These species have the same m/z value and are therefore indistinguishable in the mass spectrum. However, they can be distinguished by IM because their Ω/z will differ. For example, the charge of the Te would be four times that of the M; however, the Ω_{Te} would be smaller than four times the Ω_M because of the favorable interactions within the monomers to form tetramers. Thus, it follows that $\Omega_{Te}/4z < \Omega_{Tr}/3z < \Omega_D/2z < \Omega_M/z$. Thus, when analyzing mobility times, the species that have smallest drift times were initially assigned to those corresponding to the largest oligomer. For example, for the LMW Aβ40 spectra at m/z 2165, four contributions were detected in the drift time domain at 4.1, 5.1, 7.7, and 12.8 ms (Fig. 3b). The peaks at 4.1 and 12.8 ms were initially assigned to the highest and lowest oligomer order, respectively.

Secondly, we assigned a given oligomer order on the basis of the ^{13}C isotope distribution associated with each of the peaks separated in the mobility dimension. Applying this consideration to the m/z peak of LMW Aβ40 observed at m/z 2165, the ^{13}C isotope distribution associated with the mobility peaks detected at 12.8 and 7.7 ms were consistent with charges +2 and +4, respectively, and therefore were assigned to M +2 and D +4, respectively (Fig. 3b). The resolution of the ^{13}C isotope distribution for the mobility peaks observed at 5.1 and 4.1 ms was not sufficient for their assignation. In this case, the charge envelope in the 2D ESI-IM-MS spectrum was

analyzed. In the ESI-IM-MS spectra, we found other charge states consistent with the assignment of the peaks at 5.1 and 4.1 ms to Tr +6 and Te +8, respectively. For the former, these were Tr + 5 and Tr +7 and for the latter Te +7 (Fig. 3a).

Finally, the conformation of the ions was also taken into account for the assignment of the ESI-IM-MS peaks. Peaks that had the same m/z and the same ^{13}C isotope distribution were attributed to compact and extended forms of the same oligomer. The m/z peak of LMW Aβ40 observed at m/z 1732 showed two contributions in the drift time domain (at 4.4 and 7.4 ms), and the ^{13}C isotope distribution associated with these two mobility peaks was consistent with +5 charges, so they were assigned to compact and extended forms of the D +5, respectively (Fig. 3c). Using this analysis, all the peaks in the ESI-IM-MS spectra were assigned to specific Aβ oligomers for all four samples under study, namely LMW Aβ40 (Fig. 3), LMW_CL Aβ40 (Supplementary Fig. S3), LMW Aβ42 (Supplementary Fig. S4), and LMW_CL Aβ42 (Supplementary Fig. S5).

Supplementary Figures

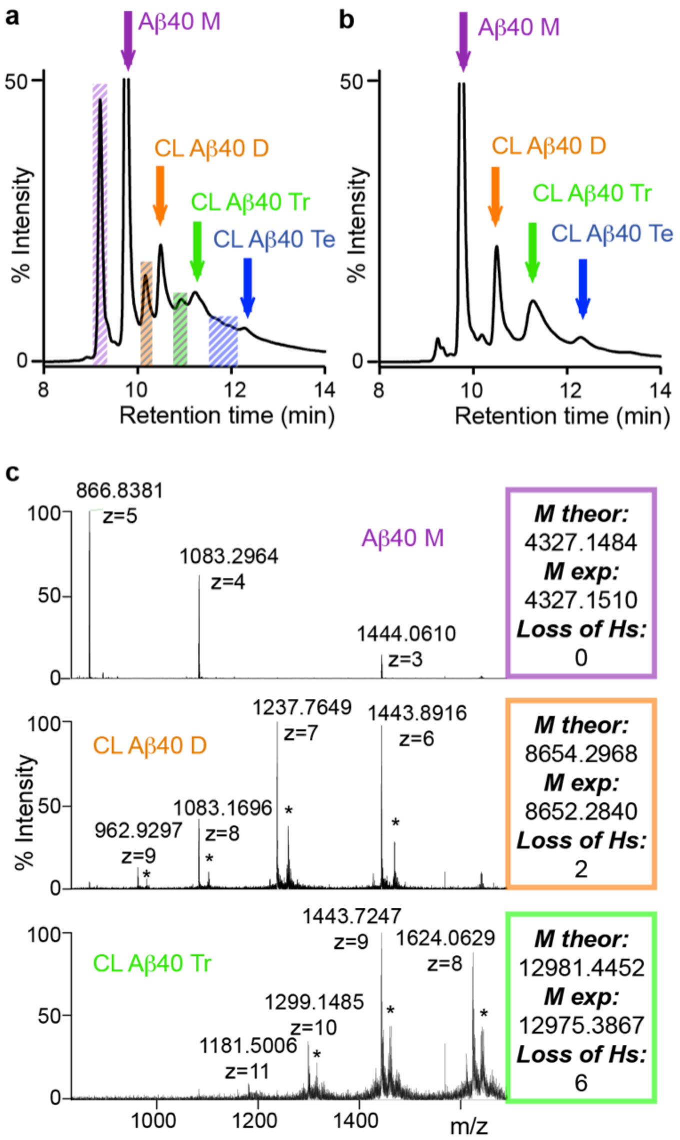

Supplementary Figure S1. Optimization of PICUP conditions to produce chemically well-defined low-n CL Aβ oligomers. RP-HPLC characterization of LMW_CL Aβ40 samples, using an Aβ/Ru(bpy)$_3^{2+}$/APS ratio of (**a**) 1:2:40 and (**b**) 1:2:5, light source at a distance of 10 cm and irradiation for 1 s. LC-HRMS characterization of LMW_CL Aβ40 samples obtained using an Aβ/Ru(bpy)$_3^{2+}$/APS ratio of 1:2:40 revealed the presence of different degrees of oxidized byproducts (Supplementary Table S1). Oxidized monomers, CL dimers, CL trimers, and CL

tetramers are indicated, respectively, as purple, orange, green, and blue dashed bars in panel (a). (**c**) LC-HRMS characterization of LMW_CL Aβ40 samples obtained using an Aβ/$Ru(bpy)_3^{2+}$/APS ratio of 1:2:5. The ESI-mass spectrum corresponding to each of the peaks detected by LC-HRMS is shown. Theoretical monoisotopic mass (M theor., considering the intact mass of the oligomer without any cross-links), experimental monoisotopic mass (M exp.), and the resulting loss of hydrogen atoms upon cross-linking (loss of Hs) for each species detected are shown next to ESI-MS spectra. The peaks labeled with an * correspond to dithiothreitol (DTT) adducts. Although the abundance of CL tetramers was low, we detected a +10 charge state consistent with the formation of four covalent bonds within them (M theor.= 17308.5936; M exp. = 17300.5311; loss of Hs = 8).

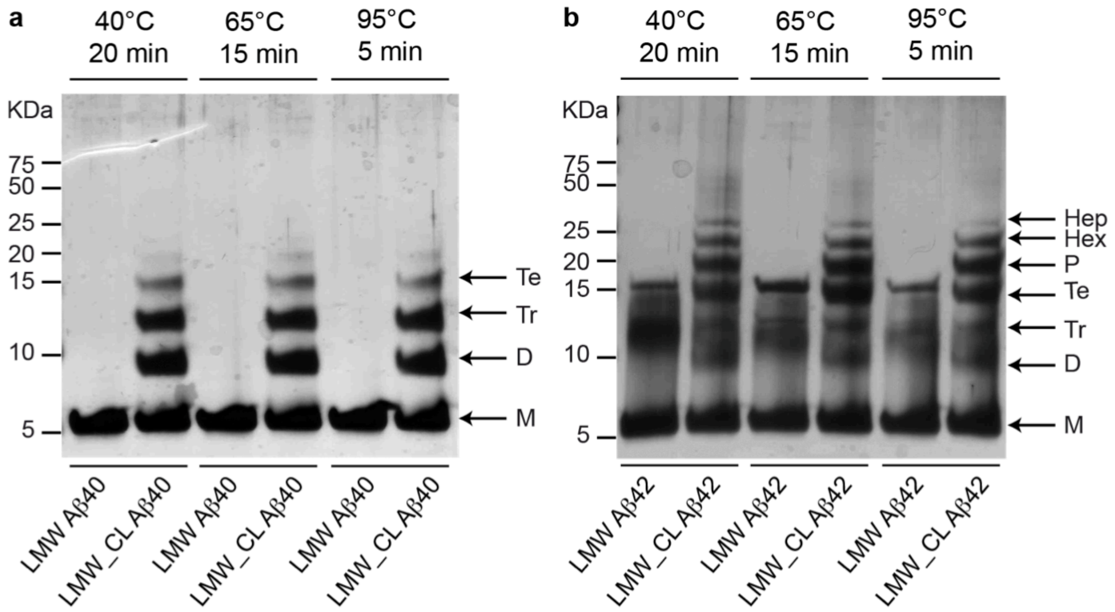

Supplementary Figure S2. Effect of incubation temperature and time on SDS-PAGE analysis of LMW and LMW_CL Aβ40 and Aβ42 samples. Characterization of (**a**) LMW and LMW_CL Aβ40 and (**b**) LMW and LMW_CL Aβ42 samples by SDS-PAGE. Samples were incubated at the indicated temperature and time prior to gel loading. LMW_CL Aβ40 and Aβ42 samples were prepared using an Aβ/$Ru(bpy)_3^{2+}$/APS ratio of 1:2:5.

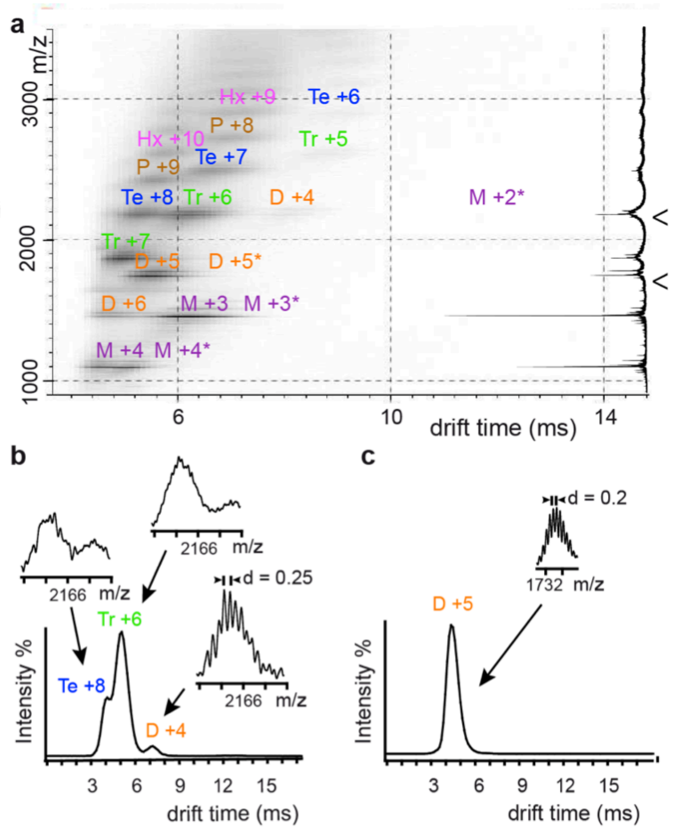

Supplementary Figure S3. ESI-IM-MS analysis of LMW_CL Aβ40. (**a**) ESI-IM-MS spectra. M = monomers, D = dimers, Tr = trimers, Te = tetramers, P = pentamers, and Hx = hexamers. The number adjacent to each aggregation state refers to the charge state of the ion. The summed m/z spectrum is shown on the right. Projections of the ESI-IM-MS spectra on the drift time axis for (**b**) m/z 2166 (M +2, D +4, Tr +6, Te +8) and (**c**) m/z 1732 (D +5), both indicated by an arrowhead in (a). For each of the detected mobility peaks, their associated m/z spectrum is also shown. Peaks were assigned on the basis of the considerations described in the Supplementary Text.

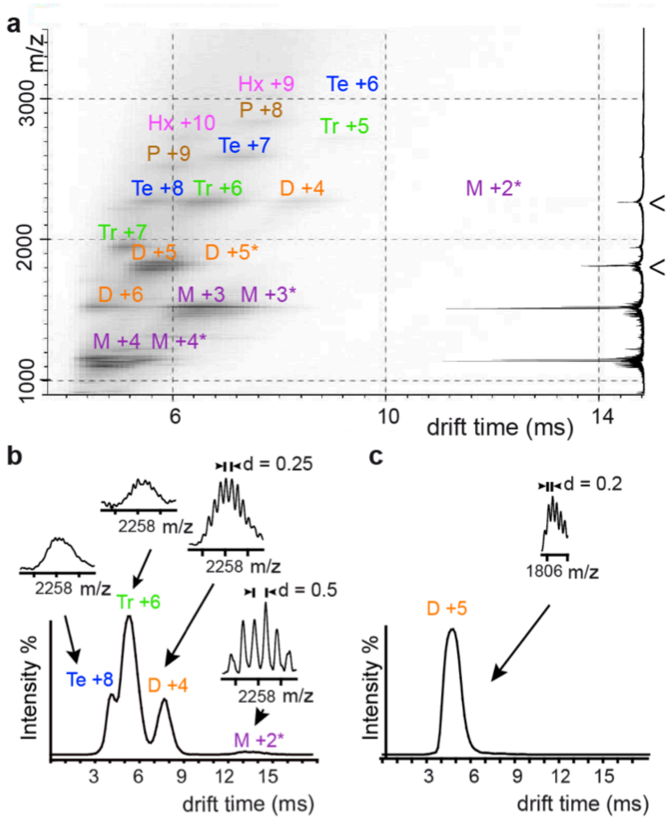

Supplementary Figure S4. ESI-IM-MS analysis of LMW Aβ42. (**a**) ESI-IM-MS spectra. M = monomers, D = dimers, Tr = trimers, Te = tetramers, P = pentamers, and Hx = hexamers. The number adjacent to each aggregation state refers to the charge state of the ion. The summed m/z spectrum is shown on the right. Projections of the ESI-IM-MS spectra on the drift time axis for (**b**) m/z 2258 (M +2, D +4, Tr +6, Te +8) and (**c**) m/z 1806 (D +5), both indicated by an arrowhead in (a). For each of the detected mobility peaks, their associated m/z spectrum is also shown. Peaks were assigned on the basis of the considerations described in the Supplementary Text.

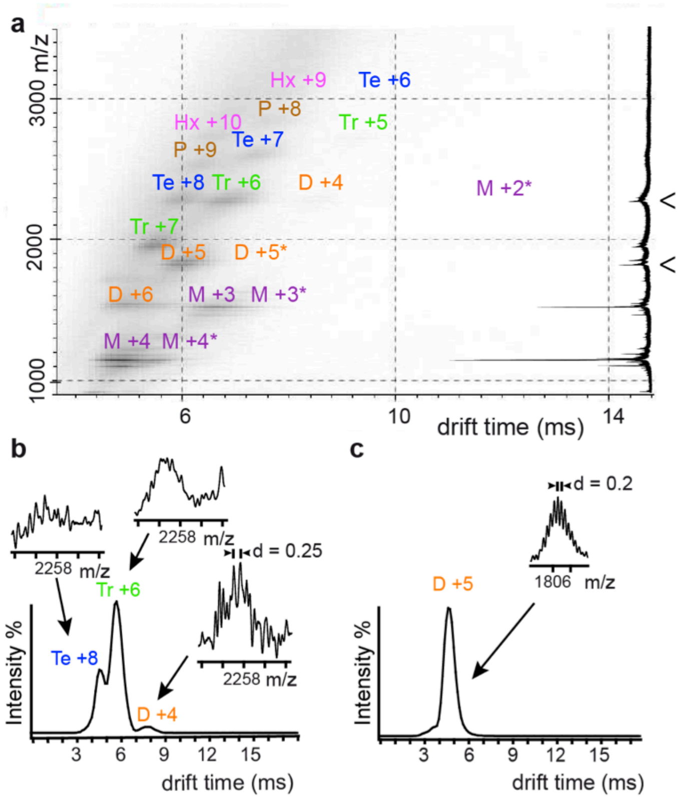

Supplementary Figure S5. ESI-IM-MS analysis of LMW_CL Aβ42. (**a**) ESI-IM-MS spectra. M = monomers, D = dimers, Tr = trimers, Te = tetramers, P = pentamers, and Hx = hexamers. The number adjacent to each aggregation state refers to the charge state of the ion. The summed m/z spectrum is shown on the right. Projections of the ESI-IM-MS spectra on the drift time axis for (**b**) m/z 2258 (M +2, D +4, Tr +6, Te +8) and (**c**) m/z 1806 (D +5), both indicated by an arrowhead in (a). For each of the detected mobility peaks, their associated m/z spectrum is also shown. Peaks were assigned on the basis of the considerations described in the Supplementary Text.

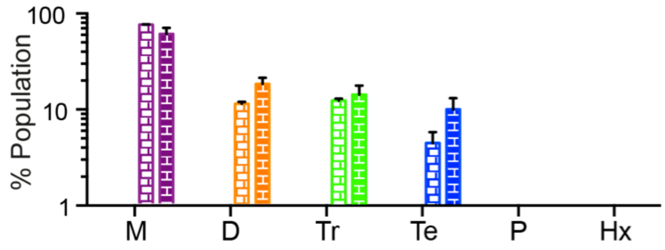

Supplementary Figure S6. Relative population of species present early during Aβ aggregation obtained from GdnHSCN-SEC analysis. M = monomers (purple), D = dimers (orange), Tr = trimers (green), Te = tetramers (blue), P = pentamers (brown), and Hex = hexamers (pink) for LMW Aβ40 (empty bars), LMW Aβ42 (filled bars), LMW_CL Aβ40 (empty brick pattern bars) and LMW_CL Aβ42 (filled bricked pattern bars) obtained from the area of all peaks detected in GdnHSCN-SEC analysis (Fig. 2c,d).

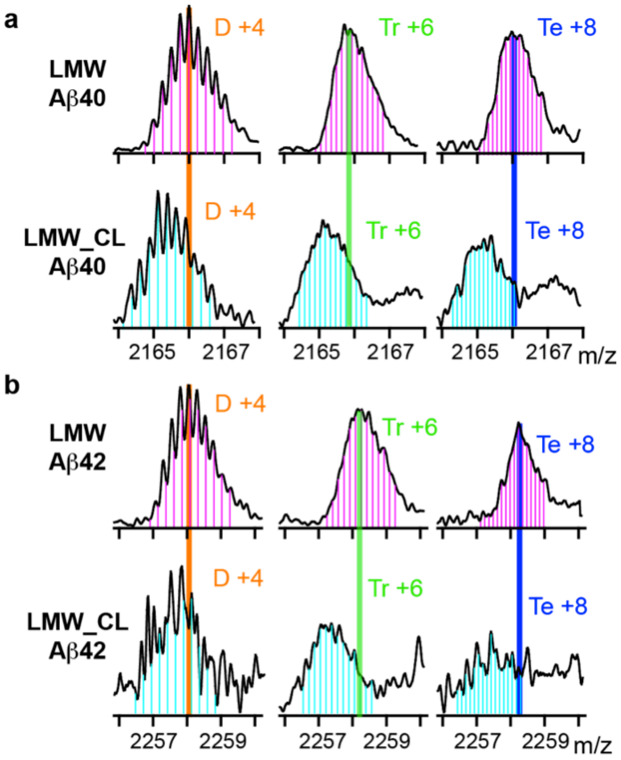

Supplementary Figure S7. ESI-IM-MS allows characterization of chemical modifications within the Aβ oligomer sequence. m/z spectra associated with mobility peaks assigned to D +4, Tr +6, and Te +8 in ESI-IM-MS spectra for (**a**) LMW and LMW_CL Aβ40 and (**b**) LMW and LMW_CL Aβ42 samples. Given that an n-order oligomer would need at least (n – 1) bonds to be covalently cross-linked, and considering that in PICUP two hydrogens are lost in the formation of each covalent bond,[1] a CL oligomer should have at least 2(n – 1) protons less that the corresponding non-covalent oligomer. The theoretical isotopic distributions, considering either the intact mass of the oligomer (isotopomers in pink) or the loss of 2(n-1) protons (isotopomers in cyan), matched those obtained experimentally for non-covalent and covalently CL Aβ oligomers, respectively.

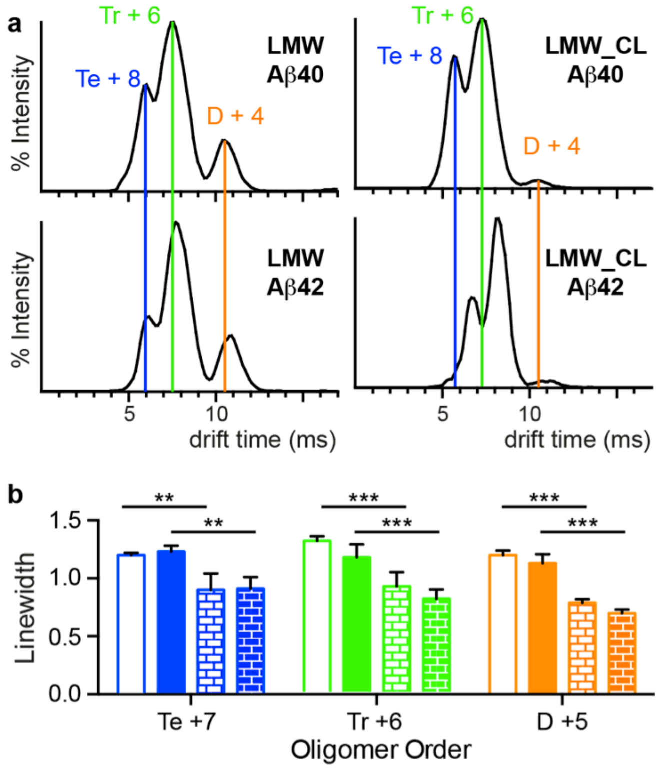

Supplementary Figure S8. Effect of cross-linking on Aβ oligomer structure and dynamics. (**a**) Projection of the ESI-IM-MS spectra on the drift time axis for m/z consistent with D +4, Tr + 6, and Te +8. (**b**) Line width from the mobility peaks associated with the most abundant charge states for each of the Aβ oligomers detected Te +7 (blue), Tr +6 (green), and D +5 (orange) for LMW Aβ40 (empty bars), LMW Aβ42 (filled bars), LMW_CL Aβ40 (empty brick-pattern bars), and LMW_CL Aβ42 (filled brick-pattern bars) samples. Data are the mean ± s.d. of three independent experiments. p-values are calculated using unpaired two-tailed Student's t-test (**$p < 0.01$ and ***$p < 0.001$). The line width corresponding to non-covalent Aβ oligomers in LMW samples was significantly larger than that of CL cross-linked ones in LMW_CL samples. This finding indicated that during the mobility experiment, each of the non-covalent Aβ oligomers sampled more conformations than their corresponding CL counterparts. Thus, our results are consistent with the general expectation that cross-linking reduces the number of accessible conformations.

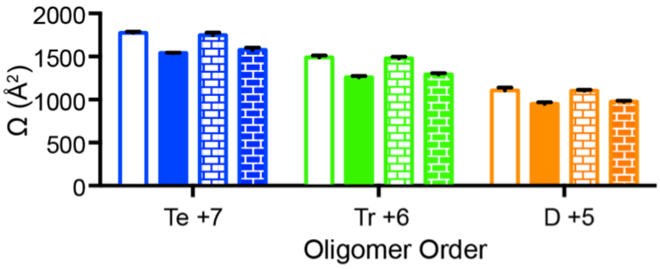

Supplementary Figure S9. The differences in Ω for Aβ40 and Aβ42 can be explained by the two extra amino acids (IA) of the latter. The calculated Ω for these two extra amino acids was 84 ± 4 Å. To determine the effect of these two amino acids on the Ω of Aβ42 oligomers relative to that of Aβ40, 84*n (where n is the order of the oligomer) was added to the Ω of Aβ40 oligomers obtained from the mobility peaks associated with the most abundant charge states (Te +7 (blue), Tr + 6 (green), and D +5 (orange)). The resulting value was compared with the Ω of Aβ42 oligomers. The bars correspond to LMW Aβ40 + 84*n (empty bars), LMW Aβ42 (filled bars), LMW_CL Aβ40 + 84*n (empty brick pattern bars), and LMW_CL Aβ42 (filled bricked pattern bars).

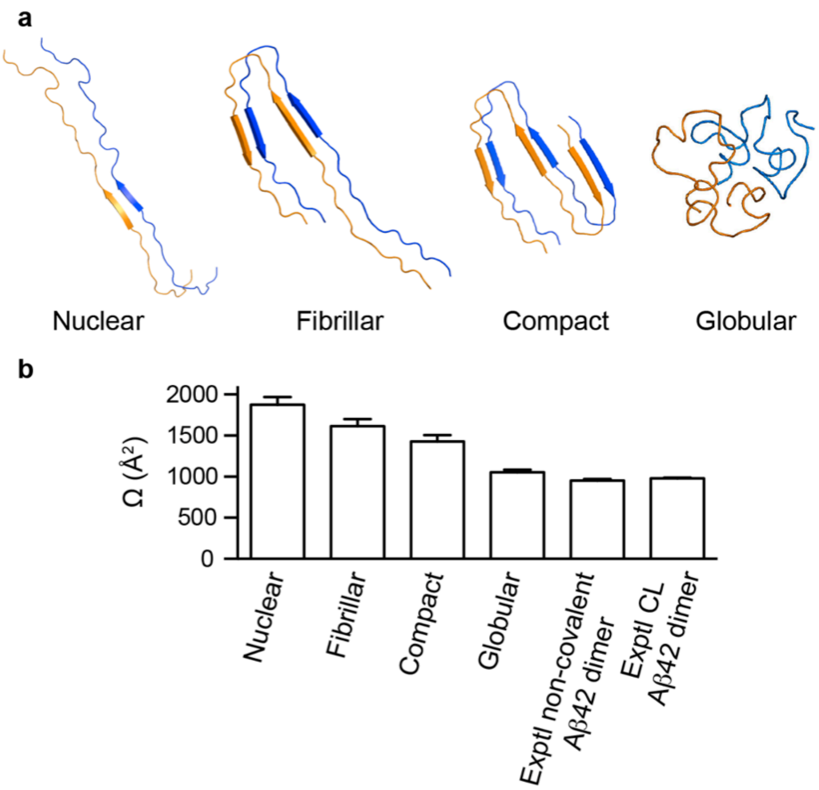

Supplementary Figure S10. The structure of LMW and LMW_CL Aβ42 dimers is globular. (**a**) Theoretical Aβ42 dimer models constructed using reported structural restraints for Aβ aggregates (nuclear[2], fibrillar[3], and compact[4,5]), or obtained from REMD simulations (globular) (**b**) Comparison of Ω values obtained from theoretical models of the dimer structures described in (a) with experimental measures obtained for the LMW Aβ42 dimers and LMW_CL Aβ42 dimers reported in Fig. 4a.

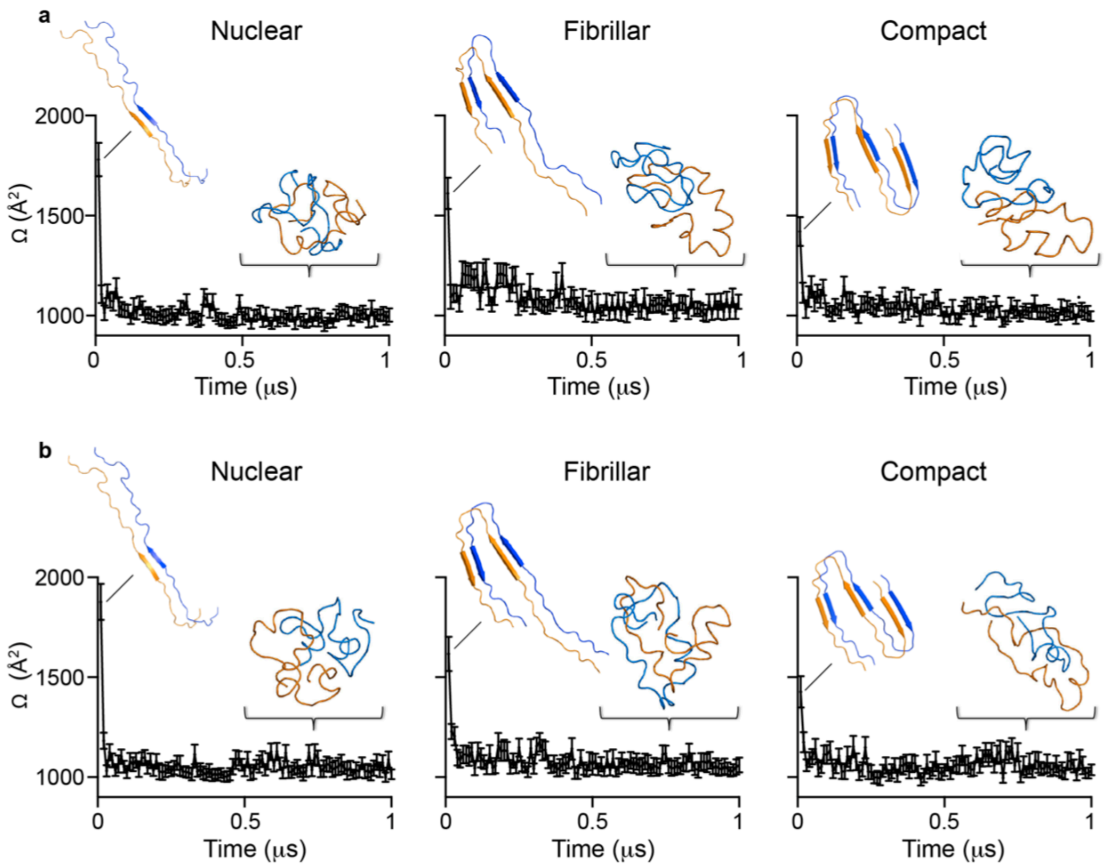

Supplementary Figure S11. Evolution to globular structures. Temporal evolution of Ω during the 1-μs REMD simulations starting from the three theoretical dimer models considered (nuclear[2], fibrillar[3], and compact[4,5]) for (**a**) Aβ40 and (**b**) Aβ42 dimers.

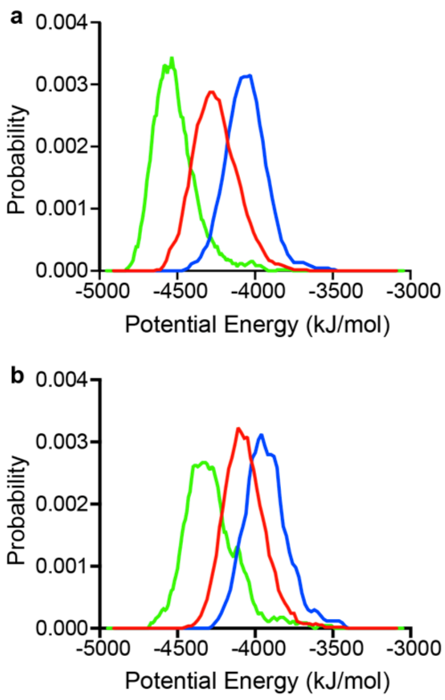

Supplementary Figure S12. Energy probability distribution. The probability distribution of the potential energy for the structures obtained from the last 500-ns trajectory of REMD simulations starting from the three theoretical dimer models (nuclear[2] [green], fibrillar[3] [blue], and compact[4,5] [red]) for (**a**) Aβ40 and for (**b**) Aβ42 dimers.

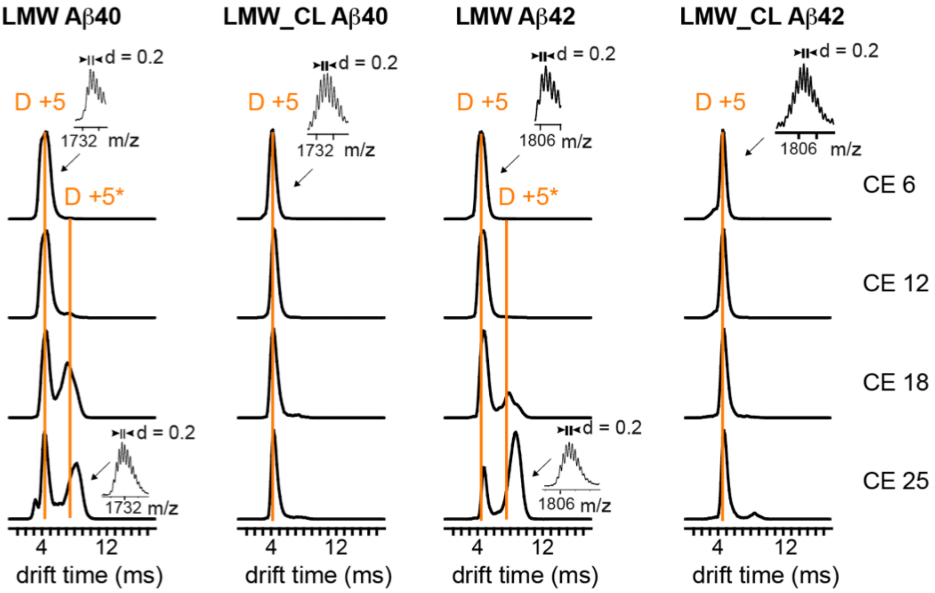

Supplementary Figure S13. Projection of the ESI-IM-MS spectra, at m/z corresponding to D +5, on the drift time axis at a range of trap collision energies (CE) for LMW and LMW_CL Aβ40 and Aβ42 samples. For each of the detected mobility peaks, their associated m/z spectrum is also shown. ^{13}C-isotope separations of 0.2 amu are consistent with charge states of +5. At high CE, the m/z peak of LMW Aβ40 and Aβ42 showed two peaks. The ^{13}C isotope distribution associated with these two peaks was consistent with +5 charges and were therefore assigned to compact and extended forms of the D +5 charge state, respectively. The * indicates that the ion has a more extended conformation.

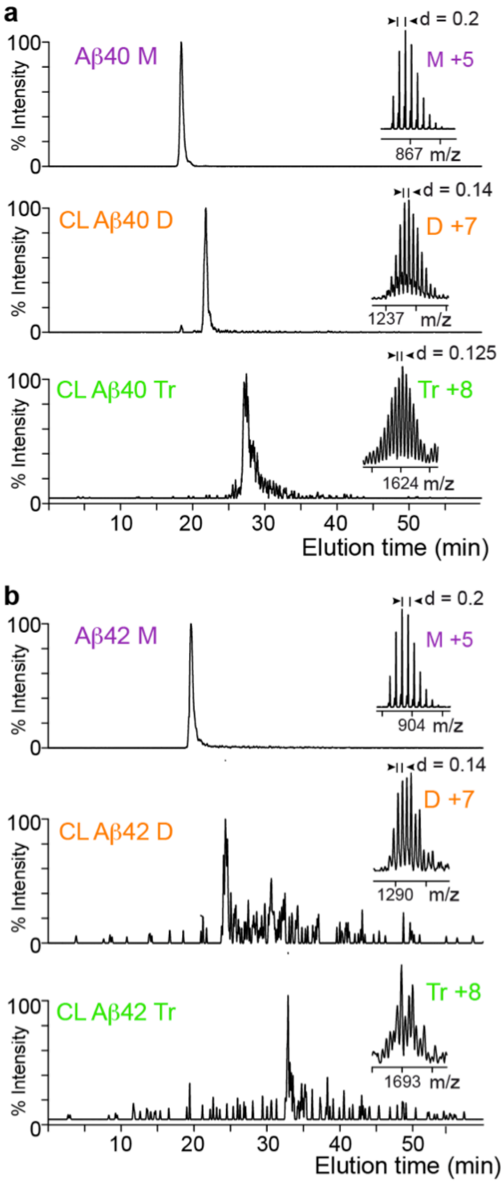

Supplementary Fig. S14. LC-HRMS analysis of LMW_CL Aβ40 and LMW_CL Aβ42. Extracted ion chromatograms for charge states corresponding to M +5, D + 7, and Tr + 8 for (a) LMW_CL Aβ40 and (b) LMW_CL Aβ42 samples analyzed by LC-HRMS. Analysis of LMW_CL Ab42 samples by HRMS was accompanied by chromatographic problems such as column plugging and high pressure, which suggested that Aβ42 CL dimers and trimers were evolving into larger aggregates during chromatography.

Supplementary Tables

Supplementary Table 1. LC-HRMS analysis of LMW_CL Aβ40 samples obtained using an Aβ/Ru(bpy)$_3^{2+}$/APS ratio of 1:2:40.

Species detected[a]	M theor[b]	M exp[c]	Loss of Hs[d]	Addition of Os[e]	Molecular formula	Error (ppm)
M	4327.1484	4327.1556	—	—	$C_{194}H_{295}O_{58}N_{53}S$	1.67
M_ox	4343.1433	4343.1530	—	1	$C_{194}H_{295}O_{59}N_{53}S$	2.24
D	8654.2968				$C_{388}H_{590}O_{116}N_{106}S_2$	
CL_D	8652.2812	8652.2937	2	—	$C_{388}H_{588}O_{116}N_{106}S_2$	1.44
CL_D_ox	8668.2761	8668.3084	2	1	$C_{388}H_{588}O_{117}N_{106}S_2$	3.73
CL_D_ox	8683.2632	8683.3026	3	2	$C_{388}H_{587}O_{118}N_{106}S_2$	4.54
Tr	12981.4452				$C_{582}H_{885}O_{174}N_{159}S_3$	
CL_Tr	12975.3982	12975.3858	6	—	$C_{582}H_{880}O_{174}N_{159}S_3$	-0.96
CL_Tr_ox	12992.4011	12992.4303	5	1	$C_{582}H_{880}O_{175}N_{159}S_3$	2.25
CL_Tr_ox	13007.3881	13007.4616	6	2	$C_{582}H_{879}O_{176}N_{159}S_3$	5.65
CL_Tr_ox	13022.3752	13022.4564	7	3	$C_{582}H_{878}O_{177}N_{159}S_3$	6.23
Te	17308.5936				$C_{776}H_{1180}O_{232}N_{212}S_4$	
CL_Te	17300.5311	17300.5607	8	—	$C_{776}H_{1172}O_{232}N_{212}S_4$	1.71
CL_Te_ox	17316.5260	17316.7115	8	1	$C_{776}H_{1172}O_{233}N_{212}S_4$	10.71
CL_Te_ox	17332.5209	17332.6004	8	2	$C_{776}H_{1172}O_{234}N_{212}S_4$	4.59
CL_Te_ox	17347.5080	17347.5448	9	3	$C_{776}H_{1171}O_{235}N_{212}S_4$	2.12
CL_Te_ox	17363.5029	17363.5949	9	4	$C_{776}H_{1171}O_{236}N_{212}S_4$	5.29

[a]Species detected: M = monomer, D = dimer, Tr = trimer, Te = tetramer, CL = cross-linked and ox = oxidized byproduct.

[b]M theor: Theoretical monoisotopic mass.

[c]M expt: Experimental monoisotopic mass.

[d]Loss of Hs: The resulting loss of hydrogen atoms upon cross-linking.

[e]Addition of Os: The resulting number of oxygen atoms caused by undesired oxidation.

Supplementary Table 2. Ω values obtained from the mobility peaks associated with the two most abundant charge states for each of the Aβ species detected.

Aggregation State	Aβ sample	Charge State	Ω (Å^2)	SD
Monomer	LMW Aβ40	4	657.29	38.15
		3	592.14	33.69
	LMW Aβ42	4	723.43	9.41
		3	615.34	18.66
	CL Aβ40	4	681.78	35.53
		3	596.90	11.42
	CL Aβ42	4	696.40	4.26
		3	629.77	4.46
Dimer	LMW Aβ40	6	970.60	28.98
		5	938.73	33.61
	LMW Aβ42	6	982.51	20.66
		5	952.17	19.85
	CL Aβ40	6	987.05	26.72
		5	936.43	10.50
	CL Aβ42	6	1017.42	22.49
		5	978.40	9.10
Trimer	LMW Aβ40	7	1183.97	43.01
		6[a]	1223.91	21.38
	LMW Aβ42	7	1234.26	27.56
		6[a]	1243.87	17.31
	CL Aβ40	7	1207.73	30.84
		6[a]	1211.15	19.30
	CL Aβ42	7	1290.33	20.79
		6[a]	1277.65	16.16
Tetramer	LMW Aβ40	8[a]	1444.94	47.59
		7	1501.30	12.96
	LMW Aβ42	8[a]	1474.56	37.41
		7	1518.36	4.73
	CL Aβ40	8[a]	1440.40	25.26
		7	1474.91	29.17
	CL Aβ42	8[a]	1562.12	20.15
		7	1556.92	26.42

[a]Ion mobility peak overlapped, drift time obtained from Gaussian fitting.

References

1 Fancy, D.A. & Kodadek, T. Chemistry for the analysis of protein-protein interactions: rapid and efficient cross-linking triggered by long wavelength light. *Proc. Natl. Acad. Sci. USA* **96**, 6020-6024 (1999).

2 Reinke, A.A., Ung, P.M., Quintero, J.J., Carlson, H.A. & Gestwicki, J.E. Chemical probes that selectively recognize the earliest Abeta oligomers in complex mixtures. *J. Am. Chem. Soc.* **132**, 17655-17657 (2010).

3 Petkova, A.T., Yau, W.M. & Tycko, R. Experimental constraints on quaternary structure in Alzheimer's beta-amyloid fibrils. *Biochemistry* **45**, 498-512 (2006).

4 Ahmed, M. *et al.* Structural conversion of neurotoxic amyloid-beta(1-42) oligomers to fibrils. *Nat. Struct. Mol. Biol.* **17**, 561-567 (2010).

5 Haupt, C. *et al.* Structural basis of beta-amyloid-dependent synaptic dysfunctions. *Angew. Chem. Int. Ed. Engl.* **51**, 1576-1579 (2012).
